# Supplementary material for: Association between atherogenic coefficient and depression in US adults: a cross-sectional study with data from National Health and Nutrition Examination Survey 2005–2018
Source: BMJ Open. 2023 Oct 29;13(10):e074001. doi: 10.1136/bmjopen-2023-074001 (PMC10619029; doi:10.1136/bmjopen-2023-074001)
Supplement: Supplementary data [file bmjopen-2023-074001supp001.pdf]

Table S1. Missing covariates of study participants (n = 32502)

Variable	Number of patients (% missing)
Age	0 (0%)
Sex	0 (0%)
Race/ethnicity	0 (0%)
Educational level	23 (0.07%)
Marital status	16 (0.04%)
Poverty-income ratio	2767 (8.51%)
Body mass index	297 (0.91%)
Alcohol intake	4661 (14.34%)
Smoking status	17 (0.05%)
Physical activity	4102 (12.62%)
Hypertension	0 (0%)
Diabetes mellitus	589 (1.81%)
Glycosylated hemoglobin	58 (0.18%)

Table S2. Absence of atherogenic coefficient in adults

**Age (y)**	**Missing number (n)**	**Total number (n)**	**Proportion (%)**
20-29	729	6029	12.09
30-39	665	6044	11.00
40-49	527	6060	8.70
50-59	524	5691	9.21
60-69	575	5974	9.63
70-79	425	3730	11.39
≥80	446	2330	19.14

Table S3. Threshold effect analysis for association of atherogenic coefficient with depression

**Outcomes**	**Depression**	***P*-value**
Model 1, β(95%)		
Linear effort model	1.04(1.02,1.07)	0.002
Model 2, β(95%)		
Infection point (K)	1.2	
K ＜1.2	0.54 (0.29,1.03)	0.059
1.2 ＞K	1.05 (1.02,1.08)	＜0.001
LLR	0.051	

Table S4. Results of collinearity detection

Model		Unstandardized Coefficients		Standardized Coefficients	t	Significance	Collinearity Statistics	
		B	Standard Error				Tolerance	VIF
1	Constant	-0.067	0.01		-6.668	0		
	AC	0.002	0.002	0.01	1.089	0.276	0.153	6.524
	HDL cholesterol	-0.002	0.006	-0.003	-0.349	0.727	0.193	5.177
	TC	-0.001	0.002	-0.002	-0.312	0.755	0.269	3.723
	HbA1c	0.001	0.001	0.004	0.973	0.33	0.936	1.068

Dependent Variable: Depression.

Abbreviations: AC, atherogenic coefficient; HDL, high-density lipoprotein; TC, total cholesterol; HbA1c, glycosylated hemoglobin.
