# Supplementary material for: Association between atherogenic coefficient and depression in US adults: a cross-sectional study with data from National Health and Nutrition Examination Survey 2005–2018
Source: BMJ Open. 2023 Oct 29;13(10):e074001. doi: 10.1136/bmjopen-2023-074001 (PMC10619029; doi:10.1136/bmjopen-2023-074001)
Supplement: Author's manuscript [file bmjopen-2023-074001.draft_revisions.pdf]

BMJ Open is committed to open peer review. As part of this commitment we make the peer review history of every article we publish publicly available.

When an article is published we post the peer reviewers' comments and the authors' responses online. We also post the versions of the paper that were used during peer review. These are the versions that the peer review comments apply to.

The versions of the paper that follow are the versions that were submitted during the peer review process. They are not the versions of record or the final published versions. They should not be cited or distributed as the published version of this manuscript.

BMJ Open is an open access journal and the full, final, typeset and author-corrected version of record of the manuscript is available on our site with no access controls, subscription charges or pay-per-view fees (http://bmjopen.bmj.com).

If you have any questions on BMJ Open's open peer review process please email info.bmjopen@bmj.com

BMJ Open

Association between atherogenic coefficient and depression in US adults: a cross-sectional study with data from National Health and Nutrition Examination Survey 2005-2018

Journal:	*BMJ Open*
Manuscript ID	bmjopen-2023-074001
Article Type:	Original research
Date Submitted by the Author:	24-Mar-2023
Complete List of Authors:	Zhang, Lu; Shandong University of Traditional Chinese Medicine<br>Yin, Jiahui; Shandong University of Traditional Chinese Medicine<br>Sun, Haiyang; Guangzhou University of Traditional Chinese Medicine<br>Yang, Jiguo; Shandong University of Traditional Chinese Medicine<br>Liu, Yuanxiang; Affiliated Hospital of Shandong University of Traditional Chinese Medicine
Keywords:	Adult psychiatry < PSYCHIATRY, Depression & mood disorders < PSYCHIATRY, Risk Factors

SCHOLARONE™
Manuscripts

I, the Submitting Author has the right to grant and does grant on behalf of all authors of the Work (as defined in the below author licence), an exclusive licence and/or a non-exclusive licence for contributions from authors who are: i) UK Crown employees; ii) where BMJ has agreed a CC-BY licence shall apply, and/or iii) in accordance with the terms applicable for US Federal Government officers or employees acting as part of their official duties; on a worldwide, perpetual, irrevocable, royalty-free basis to BMJ Publishing Group Ltd ("BMJ") its licensees and where the relevant Journal is co-owned by BMJ to the co-owners of the Journal, to publish the Work in this journal and any other BMJ products and to exploit all rights, as set out in our licence.

The Submitting Author accepts and understands that any supply made under these terms is made by BMJ to the Submitting Author unless you are acting as an employee on behalf of your employer or a postgraduate student of an affiliated institution which is paying any applicable article publishing charge ("APC") for Open Access articles. Where the Submitting Author wishes to make the Work available on an Open Access basis (and intends to pay the relevant APC), the terms of reuse of such Open Access shall be governed by a Creative Commons licence – details of these licences and which Creative Commons licence will apply to this Work are set out in our licence referred to above.

Other than as permitted in any relevant BMJ Author's Self Archiving Policies, I confirm this Work has not been accepted for publication elsewhere, is not being considered for publication elsewhere and does not duplicate material already published. I confirm all authors consent to publication of this Work and authorise the granting of this licence.

For peer review only

Association between atherogenic coefficient and depression in US adults: a cross-sectional study with data from National Health and Nutrition Examination Survey 2005-2018

Lu Zhang[a], Jiahui Yin[b], Haiyang Sun[c], Jiguo Yang[d*], Yuanxiang Liu[e*],

[a]*Shandong University of Traditional Chinese Medicine, Jinan, China.*

[b]*Shandong University of Traditional Chinese Medicine, Jinan, China.*

[c]*Guangzhou University of Traditional Chinese Medicine, Guangzhou, China.*

[d]*Shandong University of Traditional Chinese Medicine, Jinan, China.*

[e]*Affiliated Hospital of Shandong University of Traditional Chinese Medicine, Jinan, China.*

* Correspondence: Yuanxiang Liu: lyxlwtg001@163.com;

Address: No.16369 Jingshi Road, Lixia District, Jinan, China

For peer review only

ABSTRACT

Objective: There are strong comorbidities between depression and cardiovascular disease. The atherogenic coefficient (AC) is an important index that is linked to cardiovascular disease and has been suggested to play a role in depression. Therefore, we investigated the association between AC and depression among adult Americans.

Design: Cross- sectional study.

Setting: The National Health and Nutrition Examination Survey (2005-2018).

Participants: A total of 32502 participants aged 20 years or older who had complete information for AC and depression were included in this study.

Primary and secondary outcome measures: Whether the patient suffered from depression. AC values were calculated from cholesterol and high-density lipoproteins. Covariates including age, sex, race/ethnicity, body mass index, poverty-income ratio, educational level, marital status, hypertension, diabetes mellitus, alcohol intake, smoking status, physical activity, and glycosylated hemoglobin level were adjusted in multivariate logistic regression models.

Results: After adjusting for potential confounders, a single unit increase in AC was associated with a 4% increase in the prevalence of depression (hazard ratio =1.04, 95% confidence interval =1.02-1.07, *P* = 0.002). The relationship between AC and depression was more obvious in females.

Conclusions: The AC is positively associated with depression.

Keywords: atherosclerosis, depression, nutrition surveys, adult

Strengths and Limitations: 1.The quality and scale of the National Health and Nutrition Examination Survey database and the rigour of its measures ensure the statistical power and reliability of our results. 2. A wide range of sociodemographic, lifestyle and physical health covariates were adjusted for, reducing the possibility of residual confounding. 3. This study was limited by its cross- sectional design, and no causal relationships could be determined. 4. The PHQ-9 is a proven, simple, and effective tool for identifying the severity of depressive symptoms, but it is not a diagnostic tool for Major Depressive Disorder (MDD).

For peer review only

INTRODUCTION

Depression is a clinically common emotional state characterized by a persistent feeling of sadness or inability to experience pleasure, accompanied by deficits in daily functioning [1]. In 2008, the World Health Organization (WHO) ranked major depression as the third leading cause of the global burden of disease and projected that it would be the number one cause by 2030 [2]. More than 300 million people worldwide suffer from major depressive disorder (MDD) [3], and this number has increased over the past decade [4]. Depression can cause various adverse events, seriously endangering lives and global health [5,6]. Current antidepressant treatments are effective, but there are many side effects; for example, antidepressants may increase suicidal thoughts in some people [7]. Evidence supports screening for depression and providing early intervention [8]. Therefore, it is necessary to explore the factors related to depression. Abnormal lipid metabolism is often observed in patients with depression. Some studies have shown that changes in circulating lipid concentrations may be associated with depression [9]. Lipids play a role in depression via inflammation and metabolic changes. On one hand, activation of the proinflammatory response leads to a decrease in high-density lipoprotein (HDL) and phospholipids and a compensatory increase in phospholipid-rich low-density lipoprotein (LDL), which in turn slows total cholesterol (TC) metabolism and affects neurotransmitters and neural circuits that contribute to behavioral symptoms of depression [10]. On the other hand, cytokine signaling in adipose tissue, particularly tumor necrosis factor (TNF), promotes metabolic dysregulation and increases depression [11].

The pathogenesis of atherosclerosis is based on the lipid theory, and the explanation is related to excess cholesterol being the sole cause of lipid deposition in the arterial wall [12]. The atherogenic coefficient (AC) is an important index for assessing the degree of atherosclerosis, which is calculated as (TC – HDL)/HDL [13]. Nunes et al. found that AC was elevated in patients with MDD and bipolar disorder and was also associated with cardiovascular disease (CVD) [14]. AC and CVD can be controlled by statins and other cardiovascular drugs [15,16]. Exploring the role of AC

in depression may be beneficial for the treatment of depression and depression combined with CVD.

To the best of our knowledge, the role that AC plays in depression is still unclear. Therefore, we used data from the National Health and Nutrition Examination Survey (NHANES) database to explore the association of AC with depression in adults.

METHODS

Study design and participants

Data of the participants in this study were obtained from the NHANES database, a major program conducted by the Centers for Disease Control and Prevention (CDC) to assess the health and nutritional status of 5,000 adults and children in the US annually [17]. The NHANES database contains demographic, dietary, examination, laboratory, and questionnaire data. The National Center for Health Statistics (NCHS) Research Ethics Review Board (ERB) authorized the NHANES study protocols. Further information regarding the NHANES data can be obtained from its official website (http://www.cdc.gov/nchs/nhanes.htm).

Participants in our study were screened according to the following inclusion criteria: 1) aged 20 years or above, and 2) participation in laboratory tests on an empty stomach. The exclusion criteria were as follows: 1) incomplete Patient Health Questionnaire-9 (PHQ-9) and 2) no data on total cholesterol or high-density lipoprotein levels.

Assessment of depression

The PHQ-9 is used to assess depression. The PHQ-9 contains nine items that capture the frequency of depressive symptoms such as: appetite problems, fatigue, sleep difficulties, psychomotor retardation or agitation, concentration problems, lack of interest, depressed mood, feelings of worthlessness, and suicidal ideation. It is now widely accepted as an accurate and reliable method for screening depression [18,19]. Each question is scored from ‘0’ (not at all) to ‘3’ (nearly every day), with a total score of 0–27 where a score ≥10 is considered clinically relevant depression (CRD)

For peer review only

[20]. PHQ-9 sensitivity compared with semi-structured diagnostic interviews was greater than that in previous conventional meta-analyses that combined reference standards. A cutoff score of 10 or above maximized the combined sensitivity and specificity overall and for subgroups [21].

Assessment of AC

Fasting blood was drawn from individuals aged ≥ 20 years, and the blood samples were processed, stored, and shipped to the Johns Hopkins University Lipoprotein Assay Laboratory at the Ambulator-Testing Center laboratory. HDL levels were measured directly in the serum. The apolipoprotein B (apoB)-containing lipoproteins in the specimen were reacted with a blocking reagent that rendered them non-reactive with the enzymatic cholesterol reagent under the assay conditions. Reagents were purchased from Roche/Boehringer-Mannheim Diagnostics (Mannheim, Germany). The method uses sulfated alpha-cyclodextrin in the presence of Mg+2, which forms complexes with apoB-containing lipoproteins, and polyethylene glycol-coupled cholesteryl esterase and cholesterol oxidase for HDL cholesterol measurement. HDL cholesterol data collected from participants in 2005-2006 were adjusted using the following equation: corrected HDL = (Solomon Park assigned HDL value) × (participant HDL). Total cholesterol was measured enzymatically in the serum or plasma in a series of coupled reactions that hydrolyze cholesteryl esters and oxidize the 3-OH group of cholesterol. All the information can be obtained from https://wwwn.cdc.gov/Nchs/Nhanes/.

Assessment of covariates

Covariates in this study, including body mass index (BMI), alcohol intake, physical activity, and glycosylated hemoglobin, were used as continuous variables. BMI was measured as weight (kg) divided by height (m) squared (<25.0 kg/m2 indicating normal, 25.0 to <30.0 kg/m2 indicating overweight, ≥30.0 kg/m2 indicating obese). Alcohol intake (the mean alcohol intake from the first and second dietary surveys was extracted, in which alcohol intake was defined as the alcohol intake on a single day for participants who consumed alcohol on a total of one day ever). Physical activity was self-reported by participants as either inactive, moderate, or vigorous. Categorical

For peer review only

variables included age (20–40 years, 40–60 years, ≥60 years), sex (male or female), and race/ethnicity (non-Hispanic white, non-Hispanic black, Mexican American, other Hispanic, or other race/multiple races). The poverty-income ratio (PIR) was defined as the ratio of family income to poverty threshold (<1 indicating an income below the poverty threshold and ≥1 indicating an income above the poverty threshold; the latter category was further divided into two groups: 1.00 to <2.00 and ≥2.00). Education level was categorized as high school not completed, high school completed, or high school graduate and some college or associated degrees pursued. Marital status was defined as married/living with partner or widowed/divorced/separated/never married. Hypertension (HTN) (defined as systolic blood pressure ≥140 mmHg or diastolic blood pressure ≥90 mmHg) was determined using three blood pressure measurements at different times, an existing diagnosis, or evidence of an existing antihypertensive medication regimen. Diabetes mellitus (DM) was defined as either taking glucose-lowering therapies, a glycated hemoglobin (HbA1c) concentration of ≥6.5%, use of anti-diabetic medication, oral glucose tolerance test (OGTT) ≥11.1 mmol/L, fasting plasma glucose ≥7.0 mmol/L, or random blood glucose ≥11.1 mmol/L. Smoking status was defined as people who do not smoke, <100 cigarettes during lifetime; people who formerly smoked, not currently smoking but ≥100 cigarettes consumed previously; and people who smoke ≥100 cigarettes every day or some days.

Statistical analysis

The main concern was whether AC is associated with depression after adjusting for other factors that may influence depression. Continuous variables are expressed as mean ± standard deviation, and categorical variables are expressed as percentages. The $\chi 2$ test was used to compare categorical variables between groups, one-way analysis of variance was used to compare normally distributed variables between groups, and Kruskal-Wallis H test was used to compare variables with a skewed distribution between groups. Multivariate logistic regression analysis was performed to evaluate the independent association between AC and depression. The participants were divided into four groups based on AC: < 1.9310, 1.9310 to < 2.6695, 2.6695 to <

For peer review only

3.6430, and ≥ 3.6430. We used three levels of adjustment: Model 1 was adjusted for age, sex, and race/ethnicity; Model 2 was adjusted for the variables in Model 1 plus BMI, PIR, educational level, and marital status; and Model 3 was adjusted for the variables in Model 2 plus HTN, DM, alcohol intake, smoking status, physical activity, and glycosylated hemoglobin. The imputation of missing data was conducted using the missForest R package, which is a random forest-based technique that is highly computationally efficient for high-dimensional data consisting of both categorical and continuous predictors; the missing values are presented in the table (Table S1) [22].

All analyses were performed using R software (The R Foundation, Vienna, Austria) and Empower (X&Y Solutions, Boston, MA, USA). Statistical significance was defined as a two-sided *P*-value <0.05.

RESULTS

Participant characteristics

In this study, 32,502 participants were included (Figure 1). Table 1 shows the characteristics of the participants according to their AC. There were statistically significant differences in age, sex, educational level, race/ethnicity, marital status, PIR, alcohol intake, smoking status, physical activity, BMI, HTN, DM, HbA1c, cholesterol, and HDL between the different AC groups (*P* <0.05).

Participants with the lowest AC in Q1 (< 1.9310) were likely to be female, younger, more educated, married or cohabitating, wealthier, less physically active, smoked less, consumed more alcohol, had no DM or HTN, and lower HbA1c levels.

By contrast, participants with the highest AC in Q4 (>3.6430) were likely to be male, > 40 years old, more highly educated, non-Hispanic White, married or cohabitating, wealthier, consumed less alcohol, never smoked, inactive and obese, had HTN, and higher HbA1c and cholesterol levels.

For peer review only

Table 1. Characteristics of the study population, using National Health and Nutrition Examination Survey data from 2005–2018 (N = 32,502).

Characteristic	Overall	Atherogenic coefficient quartiles† Q1 (< 1.9310)	Q2 (1.9310 to < 2.6695)	Q3 (2.6695 to < 3.6430)	Q4 (≥ 3.6430)	*p*-value
Sample size, *n* (%)	32,502 (100)	8,126 (25.00)	8,124 (24.99)	8,125 (25.00)	8,127 (25.00)	
Male, *n* (%)	15,954 (49.09)	2,882 (35.47)	3,496 (43.03)	4,322 (53.19)	5,254 (64.65)	<0.001
Age, y, *n* (%)						<0.001
20 to < 40	10,857 (33.40)	3,062 (37.68)	2,753 (33.89)	2,517 (30.98)	2,525 (31.07)	
40 to <60	10 632 (32.71)	2,089 (25.71)	2,423 (29.83)	2,856 (35.15)	3,264 (40.16)	
≥60	11,013 (33.88)	2,975 (36.61)	2,948 (36.29)	2,752 (33.87)	2,338 (28.77)	
Educational level, *n* (%)						<0.001
<High school	7,841 (24.14)	1,625 (20.01)	1,816 (22.36)	2,055 (25.30)	2,345 (28.89)	
Completed high school	7,486 (23.05)	1,724 (21.23)	1,838 (22.64)	1,965 (24.19)	1,959 (24.14)	
>High school	17,152 (52.81)	4,770 (58.75)	4,466 (55.00)	4,104 (50.52)	3,812 (46.97)	
Race/ethnicity, *n* (%)						<0.001
Non-Hispanic White	14,112 (43.42)	3,516 (43.27)	3,536 (43.53)	3,461 (42.60)	3,599 (44.28)	
Non-Hispanic Black	6,713 (20.65)	2,212 (27.22)	1,803 (22.19)	1,550 (19.08)	1,148 (14.13)	
Mexican American	5,174 (15.92)	925 (11.38)	1,159 (14.27)	1,466 (18.04)	1,624 (19.98)	
Other Hispanic	3,109 (9.57)	583 (7.17)	752 (9.26)	843 (10.38)	931 (11.46)	
Other race/multiple races	3,394 (10.44)	890 (10.95)	874 (10.76)	805 (9.91)	825 (10.15)	

For peer review only

Marital status, *n* (%)						<0.001
Married/Living with partner	19,532 (60.12)	4,363 (53.71)	4,782 (58.89)	5,067 (62.42)	5,320 (65.48)	
Widowed/Divorced/Separated/ Never married	12,954 (39.88)	3,761 (46.29)	3,338 (41.11)	3,051 (37.58)	2,804 (34.52)	
PIR, *n* (%)						<0.001
< 1.00	6,103 (20.46)	1,425 (19.08)	1,484 (19.90)	1,478 (19.90)	1,716 (22.97)	
1.00 to <2.00	8,001 (26.83)	1,857 (24.86)	1,920 (25.74)	2,075 (27.94)	2,149 (28.76)	
≥2.00	15,722 (52.71)	4,188 (56.06)	4,055 (54.36)	3,873 (52.15)	3,606 (48.27)	
Alcohol intake, *g/day*, mean (SD)	7.80 (19.98)	10.29 (23.26)	7.50 (19.02)	6.59 (17.43)	6.88 (19.61)	<0.001
Smoking status, *n* (%)						<0.001
Never smoked	17,828 (54.88)	4,751 (58.50)	4,626 (56.89)	4,470 (55.03)	3,981 (49.02)	
Former smoker	7,993 (24.61)	1,888 (23.25)	2,003 (24.67)	2,099 (25.84)	2,003 (24.66)	
Current smoker	6,664 (20.51)	1,483 (18.26)	1,489 (18.34)	1,554 (19.13)	2,138 (26.32)	
Physical activity, *n* (%)						<0.001
Inactive	14,825 (25.20)	3,302 (46.44)	3,597 (51.02)	3,850 (54.11)	4,076 (57.22)	
Moderate	7,339 (25.84)	1,846 (25.96)	1,855 (26.31)	131 (26.44)	1,757 (24.66)	
Vigorous	2,094 (7.37)	6,01 (8.45)	534 (7.57)	487 (6.84)	472 (6.63)	
Both moderate and vigorous	4142 (14.58)	1362 (19.15)	1064 (15.09)	897 (12.61)	819 (11.50)	
BMI, kg/m^2, mean (SD)	29.29 (6.96)	26.59 (6.56)	28.99 (7.12)	30.36 (6.88)	31.20 (6.37)	<0.001
HTN, *n* (%)	13,940 (42.89)	3,211 (39.52)	3,414 (42.02)	3,586 (44.14)	3,729 (45.88)	<0.001
DM, *n* (%)	6,152 (19.28)	1,322 (16.72)	1,411 (17.79)	1,597 (19.97)	1,822 (22.56)	<0.001

For peer review only

HbA1c, %, mean (SD)	5.76 (1.08)	5.57 (0.86)	5.68 (0.94)	5.79 (1.06)	5.99 (1.36)	<0.001
Cholesterol, mmol/L, mean (SD)	5.00 (1.09)	4.41 (0.91)	4.76 (0.91)	5.08 (0.92)	5.76 (1.12)	<0.001
HDL cholesterol, mmol/L, mean (SD)	1.37 (0.42)	1.80 (0.43)	1.45 (0.29)	1.24 (0.23)	1.01 (0.21)	<0.001
Depression, *n* (%)	2871 (8.83)	620 (7.63)	649 (7.99)	762 (9.38)	840 (10.34)	<0.001

Abbreviations: DM, diabetes mellitus; BMI, body mass index; HbA1c, glycosylated hemoglobin; HDL, high-density lipoprotein; HTN, hypertension; PIR, poverty-income ratio (ratio of family income to poverty threshold); SD, standard deviation.

Association between AC and depression

In the fully adjusted model, we observed a linear relationship between AC and depression (Figure 2). The results of the multivariate logistic regression analysis are presented in Table 2. AC was positively correlated with depression in the crude model (odds ratio [OR]= 1.09, 95% confidence interval [CI]:1.07-1.12, *P* <0.0001). After adjusting for confounders, a significant association between AC and depression was detected in Models 1-3. In Model 3, all variables were adjusted; for every 1 unit increase in AC, the incidence of depression increased by 4% (OR = 1.04, 95% CI = 1.02–1.07, *P* = 0.002).

For peer review only

Table 2. Associations of the atherogenic coefficient with depression (n = 32,502).

	**Crude Modela**		**Model 1b**		**Model 2c**		**Model 3d**	
	**OR (95% CI)**	***p*-value**	**OR (95% CI)**	***p*-value**	**OR (95% CI)**	***p*-value**	**OR (95% CI)**	***p*-value**
Per 1 increase	1.09 (1.07,1.12)	<0.001	1.13 (1.10,1.16)	<0.001	1.08 (1.05,1.11)	<0.001	1.04 (1.02,1.07)	0.002
Quartiles								
Q1 (AC: < 1.9310)	McCarron et al. (2021)		McCarron et al. (2021)		McCarron et al. (2021)		McCarron et al. (2021)	
Q2 (AC: 1.9310 to < 2.6695)	1.05 (0.94,1.18)	0.394	1.09 (0.97,1.22)	0.148	1.00 (0.89,1.12)	0.958	0.99 (0.88,1.11)	0.836
Q3 (AC: 2.6695 to < 3.6430)	1.25 (1.12,1.40)	<0.001	1.37 (1.22,1.53)	<0.001	1.18 (1.05,1.32)	0.006	1.13 (1.01,1.28)	0.040
Q4 (AC: ≥ 3.6430)	1.40 (1.25,1.56)	<0.001	1.63 (1.46,1.83)	<0.001	1.30 (1.15,1.46)	<0.001	1.15 (1.02,1.30)	0.026
p for trend	<0.0001		<0.0001		<0.0001		0.0063	

Abbreviations: AC, atherogenic coefficient.

aModel 1: Adjusted for age, sex, and race/ethnicity.

bModel 2: Adjusted for the variables in Model 1 plus body mass index, poverty-income ratio, educational level, and marital status.

cModel 3: Adjusted for the variables in Model 2 plus hypertension, diabetes mellitus, alcohol intake, smoking status, physical activity, and glycosylated hemoglobin.

For peer review only

After adjusting for age, sex, race/ethnicity, BMI, PIR, educational level, marital status, HTN, DM, alcohol intake, smoking status, and physical activity, compared with participants in the first quartile (AC< 1.9310), the second group (1.9310 to < 2.6695, OR = 0.99, 95% CI = 0.88– 1.11, P = 0.836), the third group (2.6695 to < 3.6430, OR = 1.13, 95% CI = 1.01– 1.28, P = 0.040), and the fourth

group (≥ 3.6430, OR = 1.15, 95% CI = 1.02– 1.30, *P* = 0.026) had an increased prevalence of depression (P for trend was significant in all the models).

Furthermore, regarding the interaction between sex and the relationship between AC and depression, the relationship was more significant in females (OR = 1.07, 95% CI = 1.03–1.11, *P* for interaction = 0.036).

DISCUSSION

This cross-sectional study showed an association between AC and depression in adults in the United States. After adjusting for covariates, a positive linear relationship was found between AC and depression.

The details of the mechanism explaining the relationship between AC and depression need to be further explored, and there may be several possible explanations. Lipids and the immune system interact with one another and have a regulatory effect on each other. Dysregulated inflammation promotes susceptibility to depression [23]. Studies have shown that inflammatory cytokines produced in the periphery enter the cells of the central nervous system and can affect neurotransmitters and neural circuits, producing the behavioral symptoms of depression [24]. When T lymphocytes are activated, they not only participate in immune inflammation but also directly contribute to the development of depression when functionally impaired [25,26] Lipid peroxidation and oxidation-specific epitopes are formed, and the levels of antioxidants such as glutathione, glutathione peroxidase, and coenzyme Q10 are reduced, resulting in or aggravating oxidative stress [27,28].

When lipids are abnormal, the inflammatory and oxidative and nitrosative (IO&NS) pathway is further activated [29]. At this time, the increase in binding globin and high-sensitivity C-reactive protein (CRP) is accompanied by a significant increase in interleukin-6 (IL-6), tumor necrosis factor-α, interferon-γ, other pro-inflammatory cytokines, and immune inflammation [30]. These factors lead to defects in serotonin and melatonin through the kynurenine pathway, which is often considered to be one of the main causes of depression [31]. Activation of the IO&NS

For peer review only

pathway leads to mitochondrial dysfunction and subsequent cellular dysfunction [32]. Previous studies have linked mitochondrial dysfunction in various brain regions to depression [33].

Statins have also been shown to have antidepressant effects when co-prescribed with antidepressants [34]. Lowering the AC index of patients with mood disorders improves CVD outcomes [35]. CVD is a heart and blood vessel disease characterized by myocardial infarction, angina pectoris, heart failure, heart attack and stroke [36]. Etiological studies have shown that the presence of depression doubles the risk of developing CVD [37]. Factors that contribute towards the link between depression and cardiac outcome may include alterations in the autonomic nervous system, platelet receptors and function, coagulopathic factors, proinflammatory cytokines, endothelial function, neurohormonal factors, and genetic linkages [38] At the same time, patient compliance with antidepressant treatment is relatively poor [39]. Atherosclerosis is a chronic vascular inflammatory disease that is associated with oxidative stress and endothelial dysfunction [40]. Atherosclerosis is the underlying cause of CVD and AC is a major indicator of atherosclerosis [41]. Our results suggest that AC may play a role in depression. AC may be an indicator of the relationship between CVD and depression, and a potential target and marker for the treatment of depression or depression combined with CVD. The relevant mechanisms remain to be explored further.

Our study found increased odds of depression with increased AC in adults. This suggests that controlling AC may be beneficial for depression prevention. Sex may affect this relationship. In the subgroup analysis (Table 3), we found a stronger relationship between AC and depression in females. The synergistic effect of estrogen on cognitive and emotional functions may underlie the association between ovarian hormone fluctuations and depression in females [42]. The induction of indoleamine 2, 3-dioxygenase and deleterious effects of tryptophan catabolizing metabolites (TRYCATs) play a role in the pathophysiology of depression. Activation of IDO decreases plasma tryptophan levels and increases TRYCAT synthesis in depressed individuals. Compared to males, females showed more IDO activation and TRYCAT

For peer review only

production after immune challenge [43]. This sex difference in immune dysregulation may therefore contribute to higher levels of anxiety and depression experienced by females.

Table 3. Subgroup analysis of the effect of the atherogenic coefficient on depression (n = 32502)

Subgroup	Number of participants	OR (95% CI)	*P* for interaction
Sex, n (%)			0.036
Male	15954	1.01 (0.98, 1.05)	
Female	16548	1.07 (1.03, 1.11)	
Age, n (%)			0.463
20 – < 40	10857	1.06 (1.01, 1.10)	
40 – < 60	10632	1.02 (0.98, 1.06)	
≥ 60	11013	1.06 (1.00, 1.11)	
Educational level, n (%)			0.437
< High school	7841	1.03 (0.99, 1.08)	
Completed high school	7486	1.03 (0.97, 1.08)	
> High school	17152	1.06 (1.02, 1.11)	
Race/ethnicity, n (%)			0.084
Non-Hispanic White	14112	1.06 (1.02, 1.10)	
Non-Hispanic Black	6713	1.00 (0.94, 1.07)	
Mexican American	5174	0.97 (0.91, 1.04)	
Other Hispanic	3109	1.10 (1.03, 1.17)	
Other race/multiple races	3394	1.05 (0.96, 1.15)	
Marital status, n (%)			0.873
Married/living with partner	19532	1.04 (1.01, 1.08)	
Widowed/divorced/separated/never married	12954	1.04 (1.00, 1.08)	
PIR, n (%)			0.854
< 1.00	6103	1.04 (1.00, 1.09)	
1.00 – <2.00	8001	1.04 (1.00, 1.09)	

For peer review only

≥2.00	15722	1.03 (0.97, 1.08)	
Smoking status, n (%)			0.100
Never smoked	17828	1.08 (1.04, 1.13)	
Formerly smoked	7993	1.03 (0.98, 1.08)	
Smoke every day/ some days	6664	1.02 (0.97, 1.06)	
BMI, kg/m2, mean (SD)			0.478
Low	10729	1.07 (1.01, 1.14)	
Middle	10733	1.03 (0.98, 1.07)	
High	10743	1.04 (1.00, 1.08)	
Hypertension, n (%)			0.628
Yes	13940	1.05 (1.01, 1.09)	
No	18562	1.03 (1.00, 1.08)	
DM, n (%)			0.886
Yes	6152	1.04 (0.99, 1.09)	
No	25761	1.04 (1.01, 1.08)	
HbA1c, %, mean (SD)			0.827
Low	8737	1.06 (1.00, 1.12)	
Middle	10992	1.05 (1.01, 1.10)	
High	12715	1.04 (1.00, 1.08)	
CVD, n (%)			0.359
Yes	3571	1.08 (1.01, 1.14)	
No	28927	1.04 (1.01, 1.08)	

Abbreviations: AC, atherogenic coefficient; BMI, body mass index (calculated as weight, in kilograms, divided by the square of height, in meters); CVD, cardiovascular disease; DM, diabetes mellitus; HbA1c, glycosylated hemoglobin; HTN, hypertension; PIR, poverty-income ratio (ratio of family income to poverty threshold).

This study has some limitations. First, this was a cross-sectional study; therefore, we could not determine a causal relationship between AC and depression. Second, the PHQ-9 is a proven, simple, and effective tool for identifying the severity of depressive symptoms, but it is not a diagnostic tool for MDD.

For peer review only

CONCLUSIONS

Our research shows that in American adults, a higher AC is positively related to a higher prevalence of depression. Further studies are required to explore the underlying mechanisms and potential benefits of controlling AC levels in patients with depression.

Funding: This work was supported by the Shandong Traditional Chinese Medicine Science and Technology Development Project under Grant 2019-0178.

Competing Interests: This research received no specific grant from any funding agency in the public, commercial or not-for-profit sectors.

Author Contributions: Conceptualization, L Zhang, J Yin; methodology, L Zhang; software, J Yin; validation, H Sun, Y Liu and J Yang; formal analysis, L Zhang, J Yin, and H Sun; investigation, L Zhang; resources, L Zhang; data curation, H Sun; writing—original draft preparation, L Zhang; writing—review and editing, Y Liu and J Yang; visualization, J Yin; supervision, Y Liu and J Yang; project administration, L Zhang. All authors have read and agreed to the published version of the manuscript.

Acknowledgments: Thanks to the acupuncture-medicine team. Thanks to Prof. Liu and Prof. Yang for their careful teaching. Thanks for the help colleagues. Thanks to all who contributed to the manuscript.

Data Sharing Statement: The datasets generated and/or analyzed during the current study are available in the NHANES repository [https://www.cdc.gov/nchs/nhanes/].

References

[1] McCarron RM, Shapiro B, Rawles J, et al. Depression. Ann Intern Med. 2021;174:ITC65–ITC80.

[2] Malhi GS, Mann JJ. Depression. Lancet. 2018;392:2299–2312.

[3] Stringaris A. Editorial: what is depression? J Child Psychol Psychiatry. 2017;58:1287–1289.

For peer review only

[4] World Health Organization. Health for the world's adolescents. A second chance in the second decade. Geneva, Switzerland: World Health Organization; 2014.

[5] Butcher HK, McGonigal-Kenney M. Depression and dispiritedness in later life: a 'gray drizzle of horror' isn't inevitable. Am J Nurs. 2005;105:52–61.

[6] Hao Y, Ge H, Sun M, et al. Selecting an appropriate animal model of depression. Int J Mol Sci. 2019;20:4827.

[7] Adams SM, Miller KE, Zylstra RG. Pharmacologic management of adult depression. Am Fam Physician. 2008;77:785–792.

[8] Chilton FH, Lee TC, Willard SL, et al. Depression and altered serum lipids in cynomolgus monkeys consuming a Western diet. Physiol Behav. 2011;104:222–227.

[9] Otte C, Gold SM, Penninx BW, et al. Major depressive disorder. Nat Rev Dis Primers. 2016;2:16065.

[10] Milaneschi Y, Simmons WK, van Rossum EFC, et al. Depression and obesity: evidence of shared biological mechanisms. Mol Psychiatry. 2019;24:18–33.

[11] Nieto-Vazquez I, Fernández-Veledo S, Krämer DK, et al. Insulin resistance associated to obesity: the link TNF-alpha. Arch Physiol Biochem. 2008;114:183–194.

[12] Beverly JK, Budoff MJ. Atherosclerosis: pathophysiology of insulin resistance, hyperglycemia, hyperlipidemia, and inflammation. J Diabetes. 2020;12:102–104.

[13] Singh M, Pathak MS, Paul A. A study on atherogenic indices of pregnancy induced hypertension patients as compared to normal pregnant women. J Clin Diagn Res. 2015;9:BC05–BC08.

[14] Nunes SO, Piccoli de Melo LG, Pizzo de Castro MR, et al. Atherogenic index of plasma and atherogenic coefficient are increased in major depression and bipolar disorder, especially when comorbid with tobacco use disorder. J Affect Disord. 2015;172:55–62.

[15] Tao SH, Ren XQ, Zhang LJ, et al. Association between common cardiovascular drugs and depression. Chin Med J (Engl). 2021;134:2656–2665.

[16] Walker AJ, Kim Y, Borissiouk I, et al. Statins: neurobiological underpinnings and mechanisms in mood disorders. Neurosci Biobehav Rev. 2021;128:693–708.

[17] Fain JA. NHANES. Diabetes Educ. 2017;43:151.

For peer review only

[18] Lamers F, Jonkers CC, Bosma H, et al. Summed score of the Patient Health Questionnaire-9 was a reliable and valid method for depression screening in chronically ill elderly patients. J Clin Epidemiol. 2008;61:679–687.

[19] Leavens A, Patten SB, Hudson M, et al. Influence of somatic symptoms on Patient Health Questionnaire-9 depression scores among patients with systemic sclerosis compared to a healthy general population sample. Arthritis Care Res (Hoboken). 2012;64:1195–1201.

[20] Brooks JM, Titus AJ, Bruce ML, et al. Depression and handgrip strength among U.S. adults aged 60 years and older from NHANES 2011-2014. J Nutr Health Aging. 2018;22:938–943.

[21] Levis B, Benedetti A, Thombs BD; DEPRESsion Screening Data (DEPRESSD) Collaboration. Accuracy of Patient Health Questionnaire-9 (PHQ-9) for screening to detect major depression: individual participant data meta-analysis. BMJ. 2019;365:l1476.

[22] Stekhoven DJ, Bühlmann P. MissForest—non-parametric missing value imputation for mixed-type data. Bioinformatics. 2012;28:112–118.

[23] Beurel E, Toups M, Nemeroff CB. The bidirectional relationship of depression and inflammation: double trouble. Neuron. 2020;107:234–256.

[24] Capuron L, Lasselin J, Castanon N. Role of adiposity-driven inflammation in depressive morbidity. Neuropsychopharmacology. 2017;42:115–128.

[25] Haapakoski R, Ebmeier KP, Alenius H, et al. Innate and adaptive immunity in the development of depression: an update on current knowledge and technological advances. Prog Neuropsychopharmacol Biol Psychiatry. 2016;66:63–72.

[26] Miller AH. Depression and immunity: a role for T cells? Brain Behav Immun. 2010;24:1–8.

[27] Bartolini Gritti B, Binder CJ. Oxidation-specific epitopes are major targets of innate immunity in atherothrombosis. Hamostaseologie. 2016;36:89–96.

[28] Maes M, Mihaylova I, Kubera M, et al. Increased plasma peroxides and serum oxidized low density lipoprotein antibodies in major depression: markers that further explain the higher incidence of neurodegeneration and coronary artery disease. J Affect Disord. 2010;125:287–94.

[29] Morris G, Puri BK, Walker AJ, et al. Shared pathways for neuroprogression and somatoprogression in neuropsychiatric disorders. Neurosci Biobehav Rev. 2019;107:862–882.

For peer review only

[30] Maes M, Ruckoanich P, Chang YS, et al. Multiple aberrations in shared inflammatory and oxidative & nitrosative stress (IO&NS) pathways explain the co-association of depression and cardiovascular disorder (CVD), and the increased risk for CVD and due mortality in depressed patients. Prog Neuropsychopharmacol Biol Psychiatry. 2011;35:769–783.

[31] Gałecki P, Talarowska M. Inflammatory theory of depression. Psychiatr Pol. 2018;52:437–447.

[32] Mezzaroba L, Alfieri DF, Colado Simão AN, et al. The role of zinc, copper, manganese and iron in neurodegenerative diseases. Neurotoxicology. 2019;74:230–241.

[33] Bansal Y, Kuhad A. Mitochondrial dysfunction in depression. Curr Neuropharmacol. 2016;4:610–618.

[34] Singh D, Lippmann S. Can statins diminish depression? Prim Care Companion CNS Disord. 2018;20:17br02169.

[35] Zhang Y, Chen Y, Ma L. Depression and cardiovascular disease in elderly: current understanding. J Clin Neurosci. 2018;47:1–5.

[36] Xu C, Liang J, Xu S, et al. Increased serum levels of aldehydes are associated with cardiovascular disease and cardiovascular risk factors in adults. J Hazard Mater. 2020;400:123134.

[37] Nicholson A, Kuper H, Hemingway H. Depression as an aetiologic and prognostic factor in coronary heart disease: a meta-analysis of 6362 events among 146 538 participants in 54 observational studies. Eur Heart J. 2006;27:2763–2774.

[38] de Jonge P, Rosmalen JG, Kema IP, et al. Psychophysiological biomarkers explaining the association between depression and prognosis in coronary artery patients: a critical review of the literature. Neurosci Biobehav Rev. 2010;35:84–90.

[39] Pampallona S, Bollini P, Tibaldi G, et al. Patient adherence in the treatment of depression. Br J Psychiatry. 2002;180:104–109.

[40] Marchio P, Guerra-Ojeda S, Vila JM, et al. Targeting early atherosclerosis: a focus on oxidative stress and inflammation. Oxid Med Cell Longev. 2019;2019:8563845.

[41] Nitsa A, Toutouza M, Machairas N, et al. Vitamin D in cardiovascular disease. In Vivo. 2018;32:977–981.

For peer review only

[42] Albert KM, Newhouse PA. Estrogen, stress, and depression: cognitive and biological interactions. Annu Rev Clin Psychol. 2019;15:399–423.

[43] Maes M, Leonard BE, Myint AM, et al. The new '5-HT' hypothesis of depression: cell-mediated immune activation induces indoleamine 2,3-dioxygenase, which leads to lower plasma tryptophan and an increased synthesis of detrimental tryptophan catabolites (TRYCATs), both of which contribute to the onset of depression. Prog Neuropsychopharmacol Biol Psychiatry. 2011;35:702–721.

For peer review only

FIGURE LEGENDS

Figure 1. Flowchart for inclusion of study participants.

Figure 2. Association between AC and depression in US adults (n=4759). The black vertical line on the horizontal axis represents the caffeine distribution, the red line represents the best fit, and the difference between the dashed lines represents the 95% confidence interval. The data was adjusted for age, sex, race/ethnicity, body mass index, poverty-income ratio, educational level, marital status, hypertension, diabetes mellitus, alcohol intake, smoking status, physical activity, and glycosylated hemoglobin.

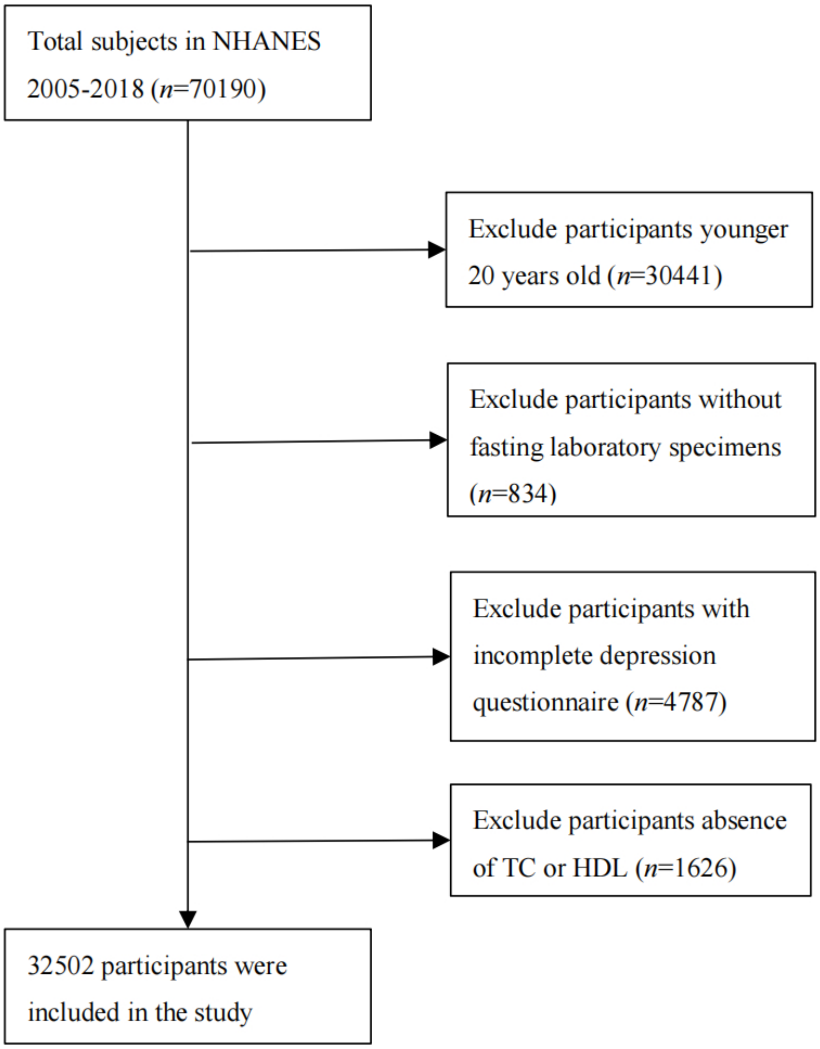

Figure 1. Flowchart for inclusion of study participants.

Flowchart for inclusion of study participants.

114x140mm (200 x 200 DPI)

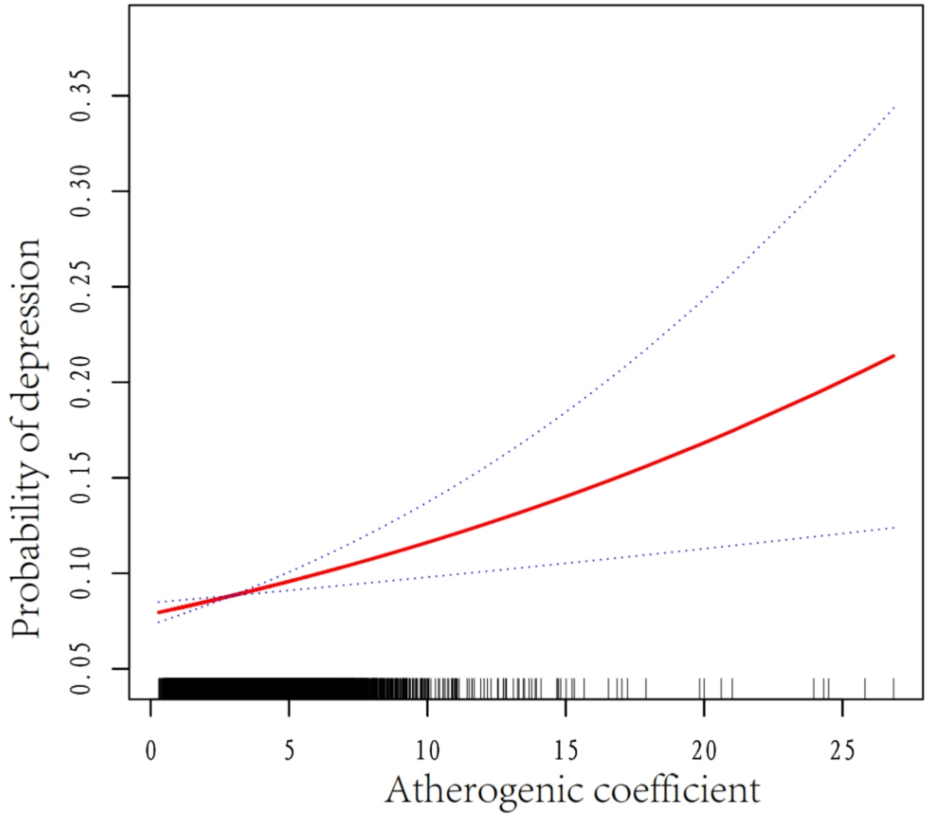

Association between AC and depression in US adults (n=4759). The black vertical line on the horizontal axis represents the caffeine distribution, the red line represents the best fit, and the difference between the dashed lines represents the 95% confidence interval. The data was adjusted for age, sex, race/ethnicity, body mass index, poverty-income ratio, educational level, marital status, hypertension, diabetes mellitus, alcohol intake, smoking status, physical activity, and glycosylated hemoglobin.

152x152mm (200 x 200 DPI)

Table S1. Missing covariates of study participants (*n* = 32502).

Variable	Number of patients (% missing)
Age	0 (0%)
Sex	0 (0%)
Race/ethnicity	0 (0%)
Educational level	23 (0.07%)
Marital status	16 (0.04%)
Poverty-income ratio	2767 (8.51%)
Body mass index	297 (0.91%)
Alcohol intake	4661 (14.34%)
Smoking status	17 (0.05%)
Physical activity	4102 (12.62%)
Hypertension	0 (0%)
Diabetes mellitus	589 (1.81%)
Glycosylated hemoglobin	58 (0.18%)

For peer review only

STROBE 2007 (v4) Statement—Checklist of items that should be included in reports of *cross-sectional studies*

Section/Topic	Item #	Recommendation	Reported on page #
**Title and abstract**	1	(*a*) Indicate the study's design with a commonly used term in the title or the abstract	2
		(*b*) Provide in the abstract an informative and balanced summary of what was done and what was found	2
**Introduction**			
Background/rationale	2	Explain the scientific background and rationale for the investigation being reported	3
Objectives	3	State specific objectives, including any prespecified hypotheses	4
**Methods**			
Study design	4	Present key elements of study design early in the paper	
Setting	5	Describe the setting, locations, and relevant dates, including periods of recruitment, exposure, follow-up, and data collection	
Participants	6	(*a*) Give the eligibility criteria, and the sources and methods of selection of participants	4
Variables	7	Clearly define all outcomes, exposures, predictors, potential confounders, and effect modifiers. Give diagnostic criteria, if applicable	5-6
Data sources/ measurement	8*	For each variable of interest, give sources of data and details of methods of assessment (measurement). Describe comparability of assessment methods if there is more than one group	6-7
Bias	9	Describe any efforts to address potential sources of bias	7
Study size	10	Explain how the study size was arrived at	4
Quantitative variables	11	Explain how quantitative variables were handled in the analyses. If applicable, describe which groupings were chosen and why	5-6
Statistical methods	12	(*a*) Describe all statistical methods, including those used to control for confounding	6-7
		(*b*) Describe any methods used to examine subgroups and interactions	5
		(*c*) Explain how missing data were addressed	7
		(*d*) If applicable, describe analytical methods taking account of sampling strategy	
		(*e*) Describe any sensitivity analyses	
**Results**			

For peer review only

Participants	13*	(a) Report numbers of individuals at each stage of study—eg numbers potentially eligible, examined for eligibility, confirmed eligible, included in the study, completing follow-up, and analysed	7-10
		(b) Give reasons for non-participation at each stage	
		(c) Consider use of a flow diagram	7
Descriptive data	14*	(a) Give characteristics of study participants (eg demographic, clinical, social) and information on exposures and potential confounders	7
		(b) Indicate number of participants with missing data for each variable of interest	7
Outcome data	15*	Report numbers of outcome events or summary measures	7
Main results	16	(*a*) Give unadjusted estimates and, if applicable, confounder-adjusted estimates and their precision (eg, 95% confidence interval). Make clear which confounders were adjusted for and why they were included	10
		(*b*) Report category boundaries when continuous variables were categorized	
		(*c*) If relevant, consider translating estimates of relative risk into absolute risk for a meaningful time period	
Other analyses	17	Report other analyses done—eg analyses of subgroups and interactions, and sensitivity analyses	14
**Discussion**			
Key results	18	Summarise key results with reference to study objectives	12
Limitations	19	Discuss limitations of the study, taking into account sources of potential bias or imprecision. Discuss both direction and magnitude of any potential bias	15
Interpretation	20	Give a cautious overall interpretation of results considering objectives, limitations, multiplicity of analyses, results from similar studies, and other relevant evidence	
Generalisability	21	Discuss the generalisability (external validity) of the study results	12-14
**Other information**			
Funding	22	Give the source of funding and the role of the funders for the present study and, if applicable, for the original study on which the present article is based	16

*Give information separately for cases and controls in case-control studies and, if applicable, for exposed and unexposed groups in cohort and cross-sectional studies.

Note: An Explanation and Elaboration article discusses each checklist item and gives methodological background and published examples of transparent reporting. The STROBE checklist is best used in conjunction with this article (freely available on the Web sites of PLoS Medicine at http://www.plosmedicine.org/, Annals of Internal Medicine at http://www.annals.org/, and Epidemiology at http://www.epidem.com/). Information on the STROBE Initiative is available at www.strobe-statement.org.

For peer review only

BMJ Open

Association between atherogenic coefficient and depression in US adults: a cross-sectional study with data from National Health and Nutrition Examination Survey 2005-2018

Journal:	*BMJ Open*
Manuscript ID	bmjopen-2023-074001.R1
Article Type:	Original research
Date Submitted by the Author:	06-Sep-2023

Complete List of Authors:	Zhang, Lu; Shandong University of Traditional Chinese Medicine<br>Yin, Jiahui; Shandong University of Traditional Chinese Medicine<br>Sun, Haiyang; Guangzhou University of Chinese Medicine<br>Yang, Jiguo; Shandong University of Traditional Chinese Medicine<br>Liu, Yuanxiang; Affiliated Hospital of Shandong University of Traditional Chinese Medicine

**Primary Subject Heading**:	Mental health
Secondary Subject Heading:	Health services research
Keywords:	Risk Factors, Adult psychiatry < PSYCHIATRY, Depression & mood disorders < PSYCHIATRY, Lipid disorders < DIABETES & ENDOCRINOLOGY

SCHOLARONE™ Manuscripts

I, the Submitting Author has the right to grant and does grant on behalf of all authors of the Work (as defined in the below author licence), an exclusive licence and/or a non-exclusive licence for contributions from authors who are: i) UK Crown employees; ii) where BMJ has agreed a CC-BY licence shall apply, and/or iii) in accordance with the terms applicable for US Federal Government officers or employees acting as part of their official duties; on a worldwide, perpetual, irrevocable, royalty-free basis to BMJ Publishing Group Ltd ("BMJ") its licensees and where the relevant Journal is co-owned by BMJ to the co-owners of the Journal, to publish the Work in this journal and any other BMJ products and to exploit all rights, as set out in our licence.

The Submitting Author accepts and understands that any supply made under these terms is made by BMJ to the Submitting Author unless you are acting as an employee on behalf of your employer or a postgraduate student of an affiliated institution which is paying any applicable article publishing charge ("APC") for Open Access articles. Where the Submitting Author wishes to make the Work available on an Open Access basis (and intends to pay the relevant APC), the terms of reuse of such Open Access shall be governed by a Creative Commons licence – details of these licences and which Creative Commons licence will apply to this Work are set out in our licence referred to above.

Other than as permitted in any relevant BMJ Author's Self Archiving Policies, I confirm this Work has not been accepted for publication elsewhere, is not being considered for publication elsewhere and does not duplicate material already published. I confirm all authors consent to publication of this Work and authorise the granting of this licence.

For peer review only

Association between atherogenic coefficient and depression in US adults: a cross-sectional study with data from National Health and Nutrition Examination Survey 2005-2018

Lu Zhang[a], Jiahui Yin[b], Haiyang Sun[c], Jiguo Yang[d*], Yuanxiang Liu[e*]
[a]*Shandong University of Traditional Chinese Medicine, Jinan, China.*
[b]*Shandong University of Traditional Chinese Medicine, Jinan, China.*
[c]*Guangzhou University of Chinese Medicine, Guangzhou, China.*
[d]*Shandong University of Traditional Chinese Medicine, Jinan, China.*
[e]*Affiliated Hospital of Shandong University of Traditional Chinese Medicine, Jinan, China.*
* Correspondence: Yuanxiang Liu: lyxlwtg001@163.com;
Address: No.16369 Jingshi Road, Lixia District, Jinan, China

For peer review only - http://bmjopen.bmj.com/site/about/guidelines.xhtml

ABSTRACT

Objective: The pathogenesis of depression is related to immune inflammatory response. AC is an important indicator of lipid abnormalities, which can lead to immune inflammatory responses. However, no study has investigated the relationship between AC and depression in adult Americans. Therefore, we investigated this relationship.

Design: This study used a cross-sectional design.

Setting: The National Health and Nutrition Examination Survey (2005-2018) data were used for this study.

Participants: A total of 32502 participants aged 20 years or older who had complete information for AC and depression were included in this study.

Primary and secondary outcome measures: Depressive symptoms were assessed using the nine-item version of the Patient Health Questionnaire (PHQ-9), with a cutoff point of 9/10 indicating likely depression cases. Weighted logistic regression analyses and the smooth curve fittings were performed to explore the association between AC and depression.

Results: After adjusting for potential confounders, a single unit increase in AC was associated with a 3% increase in the prevalence of depression (hazard ratio =1.03, 95% confidence interval =1.00-1.06, *P* = 0.039). The relationship between AC and depression was more obvious in females.

Conclusions: The AC is positively associated with depression.

Strengths and Limitations:

- The quality and scale of the National Health and Nutrition Examination Survey database ensured our results' statistical power and reliability.
- A wide range of sociodemographic, lifestyle, and physical health covariates were adjusted to reduce residual confounding.
- Its cross-sectional design limited this study, and no causal relationships could be determined.
- The PHQ-9 is a proven, simple, and effective tool for identifying the severity of depressive symptoms, but it is not a diagnostic tool for major depressive disorder (MDD).

For peer review only - http://bmjopen.bmj.com/site/about/guidelines.xhtml

For peer review only

INTRODUCTION

Depression is a clinically common emotional state characterized by persistent sadness or inability to experience pleasure, accompanied by deficits in daily functioning [1]. In 2008, the World Health Organization (WHO) ranked major depression as the third leading cause of the global disease burden and projected that it would be the number one cause by 2030 [2]. More than 300 million people worldwide suffer from major depressive disorder (MDD) [3], affecting about 8% of adults in the US [4]. Depression can cause various adverse events, seriously endangering lives and global health [5,6]. Current antidepressant treatments are effective, but there are many side effects; for example, antidepressants may increase suicidal thoughts in some people [7]. Evidence supports screening for depression and providing early intervention [8]. Therefore, it is necessary to explore the factors related to depression.

Abnormal lipid metabolism leads to many pathological changes. Firstly, activation of the pro-inflammatory response leads to a decrease in high-density lipoprotein (HDL) and phospholipids and a compensatory increase in phospholipid-rich low-density lipoprotein (LDL), which in turn slows total cholesterol (TC) metabolism and affects neurotransmitters and neural circuits [9]. However, cytokine signaling in adipose tissue, particularly tumor necrosis factor (TNF), promotes metabolic dysregulation [10]. In addition, some studies have shown that changes in circulating lipid concentrations may be associated with depression [11]. Abnormal lipids are involved in the formation of atherosclerosis. The pathogenesis of atherosclerosis is based on the lipid theory, and the explanation is related to excess cholesterol being the sole cause of lipid deposition in the arterial wall [12]. Atherosclerosis can cause cardiovascular disease (CVD), stroke, etc., often co-morbidities with depression.

The atherogenic coefficient (AC) is an important index for assessing the degree of atherosclerosis, calculated as (TC – HDL)/HDL [13]. Nunes et al. found that AC was elevated in patients with MDD and bipolar disorder[14]. AC and depression can be controlled using statins and other cardiovascular drugs [15,16].

For peer review only - http://bmjopen.bmj.com/site/about/guidelines.xhtml

Exploring the role of AC in depression may be beneficial for treating depression and its complications. Therefore, we used data from the National Health and Nutrition Examination Survey (NHANES) database to explore the association of AC with depression in adults.

METHODS

Study design and participants

Data of the participants in this study were obtained from the NHANES database, a major program conducted by the Centers for Disease Control and Prevention (CDC) to assess the health and nutritional status of 5,000 adults and children in the US annually [17]. The NHANES database contains demographic, dietary, examination, laboratory, and questionnaire data. The National Center for Health Statistics (NCHS) Research Ethics Review Board (ERB) authorized the NHANES study protocols. Further information regarding the NHANES data can be obtained from its official website (http://www.cdc.gov/nchs/nhanes.htm).

Participants in our study were screened according to the following inclusion criteria: 1) aged 20 years or above and 2) participation in laboratory tests on an empty stomach. The exclusion criteria were: 1) incomplete Patient Health Questionnaire-9 (PHQ-9) and 2) no data on total cholesterol or high-density lipoprotein levels.

Assessment of depression

The PHQ-9 is used to assess depression. The PHQ-9 contains nine items that capture the frequency of depressive symptoms: appetite problems, fatigue, sleep difficulties, psychomotor retardation or agitation, concentration problems, lack of interest, depressed mood, feelings of worthlessness, and suicidal ideation. It is now widely accepted as an accurate and reliable method for screening depression [18,19]. Each question is scored from ‘0’ (not at all) to ‘3’ (nearly every day), with a total score of 0–27, where a score ≥10 is considered clinically relevant depression (CRD) [20]. PHQ-9 sensitivity compared with semi-structured diagnostic interviews was greater than previous conventional meta-analyses that combined reference standards. A 10- or

For peer review only - http://bmjopen.bmj.com/site/about/guidelines.xhtml

above cutoff score maximized the overall sensitivity and specificity for subgroups [21].

Assessment of AC

Fasting blood was drawn from individuals aged ≥ 20 years, and the blood samples were processed, stored, and shipped to the Johns Hopkins University Lipoprotein Assay Laboratory at the Ambulator-Testing Center laboratory. HDL levels were measured directly in the serum. The apolipoprotein B (apo B)-containing lipoproteins in the specimen were reacted with a blocking reagent that rendered them non-reactive with the enzymatic cholesterol reagent under the assay conditions. Reagents were purchased from Roche/Boehringer-Mannheim Diagnostics (Mannheim, Germany). The method uses sulfated alpha-cyclodextrin in the presence of Mg^{+2}, which forms complexes with apoB-containing lipoproteins and polyethylene glycol-coupled cholesteryl esterase and cholesterol oxidase for HDL cholesterol measurement. HDL cholesterol data collected from participants in 2005-2006 were adjusted using the following equation: corrected HDL = (Solomon Park assigned HDL value) × (participant HDL). Total cholesterol was measured enzymatically in the serum or plasma in a series of coupled reactions that hydrolyze cholesteryl esters and oxidize the 3-OH cholesterol group. All the information can be obtained from https://wwwn.cdc.gov/Nchs/Nhanes/.

Assessment of covariates

Covariates in this study, including body mass index (BMI), alcohol intake, physical activity, and glycosylated hemoglobin, were used as continuous variables. BMI was measured as weight (kg) divided by height (m) squared with <25.0 kg/m2 indicating normal, 25.0 to <30.0 kg/m2 indicating overweight, ≥30.0 kg/m2 indicating obesity. Alcohol intake was determined by extracting the mean alcohol intake from the first and second dietary surveys, considering a single day's intake for participants who consumed alcohol at least once. Physical activity was self-reported by participants as either inactive, moderate, or vigorous. The study considered CVD to include coronary heart disease, congestive heart failure, heart attack, stroke, and angina. Categorical variables included age (20–40 years, 40–60 years, ≥60 years), sex (male or female),

For peer review only - http://bmjopen.bmj.com/site/about/guidelines.xhtml

and race/ethnicity (non-Hispanic white, non-Hispanic black, Mexican American, other Hispanic, or other race/multiple races). The poverty-income ratio (PIR) was defined as the ratio of family income to poverty threshold (<1 indicating an income below the poverty threshold and ≥1 indicating an income above the poverty threshold. The latter category was further classified into two groups: 1.00 to <2.00 and ≥2.00). Education level was categorized as high school not completed, high school completed, or high school graduate and some college or associated degrees pursued. Marital status was defined as married/living with a partner or widowed/divorced/separated/never married. Hypertension (HTN) (defined as systolic blood pressure ≥140 mmHg or diastolic blood pressure ≥90 mmHg) was determined using three blood pressure measurements at different times, an existing diagnosis, or evidence of an existing antihypertensive medication regimen. Diabetes mellitus (DM) was defined as either taking glucose-lowering therapies, a glycated hemoglobin (HbA1c) concentration of ≥6.5%, use of anti-diabetic medication, oral glucose tolerance test (OGTT) ≥11.1 mmol/L, fasting plasma glucose ≥7.0 mmol/L, or random blood glucose ≥11.1 mmol/L. Smoking status was categorized as non-smokers (smoked <100 cigarettes in a lifetime), former smoker (not currently smoking but have consumed ≥100 cigarettes previously), and current smoker (smoking at least ≥100 cigarettes every day or some days). The use of antidepressants, anxiolytics, sedatives, and hypnotics was were divided into use or non-use through questionnaires.

Statistical analysis

The main concern was whether AC is associated with depression after adjusting for other factors that may influence depression. Continuous variables are expressed as mean ± standard deviation, and categorical variables are expressed as percentages. The weighted $\chi 2$ test was used to compare categorical variables between groups, a one-way analysis of variance was used to compare normally distributed variables between groups, and the Kruskal-Wallis H test was used to compare variables with a skewed distribution between groups. Weighted multivariate logistic regression analysis evaluated the independent association between AC and depression. The

For peer review only - http://bmjopen.bmj.com/site/about/guidelines.xhtml

participants were categorized into four groups based on AC: < 1.9310, 1.9310 to < 2.6695, 2.6695 to < 3.6430, and ≥ 3.6430. We used three levels of adjustment: Model 1 was adjusted for age, sex, and race/ethnicity; Model 2 was adjusted for the variables in Model 1 plus BMI, PIR, educational level, and marital status; and Model 3 was adjusted for the variables in Model 2 plus HTN, DM, alcohol intake, smoking status, physical activity, and glycosylated hemoglobin. The imputation of missing data was conducted using the missForest R package. This random forest-based technique is highly computationally efficient for high-dimensional data of categorical and continuous predictors [22]. The missing values are presented in the table (Table S1).

All analyses were performed using R software (The R Foundation, Vienna, Austria) and Empower (X&Y Solutions, Boston, MA, USA). Statistical significance was defined as a two-sided *P*-value <0.05.

Patient and public involvement

None.

RESULTS

Participant characteristics

In this study, 32,502 participants were included (Figure 1). Table 1 shows the characteristics of the participants according to their AC. There were statistically significant differences in age, sex, educational level, race/ethnicity, marital status, PIR, alcohol intake, smoking status, physical activity, BMI, HTN, DM, HbA1c, cholesterol, and HDL between the different AC groups (P <0.05). In addition, there were no significant differences in the use of antidepressants, anxiolytics, sedatives, and hypnotics in participants with CVD ($p > 0.05$). Covariates with $P < 0.05$ in univariate analysis were included for further analysis.

Participants in the lowest AC in Q1 (< 1.9310) were likely to be female, younger, more educated, married or cohabitating, non-Hispanic White, wealthier, less physically active, smoked less, consumed more alcohol, had no DM or HTN, higher HDL levels, lower BMI, lower HbA1c levels, and TC levels.

For peer review only - http://bmjopen.bmj.com/site/about/guidelines.xhtml

In contrast, participants with the highest AC in Q4 (>3.6430) were likely to be male, middle-aged, more highly educated, non-Hispanic White, married or cohabitating, wealthier, consumed less alcohol, never smoked, inactive and obese, had HTN, lower HDL levels, higher BMI, higher HbA1c and TC levels.

Table 1. Characteristics of the study population, using National Health and Nutrition Examination Survey data from 2005–2018 (N = 32,502), Weighted

Characteristic	Overall	Atherogenic coefficient quartiles† Q1 (< 1.9310)	Q2 (1.9310 to < 2.6695)	Q3 (2.6695 to < 3.6430)	Q4 (≥ 3.6430)	*p*-value
Sex (%)						<0.001
Male	48.78 (48.21,49.35)	32.75 (31.33, 34.21)	42.40 (40.95, 43.87)	54.04 (52.56,55.52)	66.36 (65.00,67.70)	
Female	51.22 (50.65, 51.79)	67.25 (65.79, 68.67)	57.60 (56.13, 59.05)	45.96 (44.48, 47.44)	33.64 (32.30, 35.00)	
Age (%)						<0.001
20 to < 40	35.93 (34.76, 37.12)	40.78 (38.93, 42.66)	36.40 (34.73, 38.10)	33.15 (31.58, 34.76)	33.30 (31.69, 34.95)	
40 to <60	37.75 (36.83, 38.67)	29.54 (27.96, 31.18)	35.21 (33.59, 36.87)	40.86 (39.37,42.37)	45.58 (43.89, 47.29)	
≥60	26.32 (25.26.27.40)	29.67 (28.04, 31.36)	28.39 (26.74, 30.09)	25.99 (24.53, 27.50)	21.12 (19.78,22.52)	
Educational level (%)						<0.001
<High school	15.44 (14.36, 16.58)	12.40 (11.24, 13.65)	14.04 (12.70, 15.49)	16.18 (14.78, 17.67)	19.24 (17.90, 20.65)	
Completed high school	23.30 (22.35, 24.27)	20.49 (19.12, 21.93)	22.16 (20.82, 23.56)	24.87 (23.59,26.20)	25.75 (24.17, 27.40)	
>High school	61.26 (59.57, 62.92)	67.11 (65.08, 69.08)	63.80 (61.69, 65.86)	58.95 (56.99, 60.89)	55.02 (52.92, 57.09)	
Race/ethnicity (%)						<0.001
Non-Hispanic White	68.41 (65.86, 70.86)	69.02 (66.42, 71.51)	68.41 (65.87, 70.84)	68.23 (65.19, 71.72)	67.98 (65.01, 70.81)	
Non-Hispanic Black	10.50 (9.27,11.88)	13.76 (12.07, 15.64)	11.32 (9.98, 12.81)	9.65 (8.42, 11.03)	7.20 (6.22, 8.32)	
Mexican American	8.46 (7.22, 9.89)	6.06 (5.10, 7.18)	7.53 (6.34, 8.93)	9.50 (8.04, 11.20)	10.82 (9.06, 12.86)	
Other Hispanic	5.44 (4.65,6.35)	4.27 (3.54, 5.13)	5.22 (4.40, 6.18)	5.83 (4.87, 6.97)	6.47 (5.42, 7.70)	
Other races/multiple races	7.18 (6.50, 7.93)	6.89 (6.08, 7.81)	7.52 (6.63, 8.51)	6.79 (5.96, 7.73)	7.54 (6.67, 8.50)	
Marital status (%)						<0.001
Married/Living with a partner	64.04 (62.84, 65.22)	59.25 (57.29, 61.17)	62.92 (61.31, 64.54)	66.06 (64.48, 67.61)	68.04 (66.58,69.46)	
Widowed/Divorced/Separated/Never married	35.96 (34.78, 37.16)	40.75 (38.83, 42.71)	37.08 (35.49, 38.69)	33.94 (32.39, 35.52)	31.96 (30.54, 33.42)	
PIR (%)						<0.001
< 1.00	13.58 (12.64, 14.57)	12.69 (11.51, 13.97)	13.36 (12.18, 14.64)	12.77 (11.70, 13.92)	15.51 (14.13, 17.00)	
1.00 to <2.00	20.31 (19.33, 21.32)	18.88 (17.52, 20.31)	19.48 (18.36, 20.65)	21.07 (19.63, 22.60)	21.83 (20.18, 23.57)	
≥2.00	66.12 (64.45, 67.74)	68.44 (66.44, 70.37)	67.16 (65.18, 69.08)	66.15 (64.30, 67.96)	62.66 (60.14, 65.12)	
Alcohol intake (g/day)	9.38 (8.90,9.87)	12.33 (11.43, 13.24)	8.91 (8.22, 9.61)	7.84 (7.19, 8.49)	8.43 (7.62, 9.25)	<0.001

For peer review only

For peer review only - http://bmjopen.bmj.com/site/about/guidelines.xhtml

Smoking status (%)						<0.001
Non-smoker	54.76 (53.63, 55.88)	58.23 (56.66, 59.78)	56.97 (55.26, 58.66)	54.94 (53.42, 56.45)	48.79 (47.25, 50.33)	
Former smoker	25.13 (24.28, 26.00)	24.55 (23.30, 25.85)	24.60 (23.23, 26.02)	26.12 (24.65, 27.66)	25.26 (24.00, 26.56)	
Current smoker	20.11 (19.24, 21.01)	17.22 (16.03, 18.48)	18.44 (17.19, 19.75)	18.94 (17.85, 20.08)	25.95 (24.45,27.51)	
Physical activity (%)						<0.001
Inactive	45.81 (44.18, 47.45)	39.32 (36.97, 41.72)	44.08 (41.77, 46.42)	47.93 (46.10, 49.77)	52.15 (50.11, 54.17)	
Moderate	28.01 (26.94, 29.11)	27.48 (25.78, 29.25)	28.01 (26.30, 29.78)	29.11 (27.52, 30.76)	27.43 (25.81, 29.11)	
Vigorous	8.05 (7.55, 8.59)	8.86 (7.84, 9.99)	8.74 (7.74, 9.85)	7.25 (6.40, 8.20)	7.36 (6.37, 8.48)	
Both moderate and vigorous	18.13 (116.98, 19.34)	24.34 (22.46, 26.33)	19.17 (17.54, 20.93)	15.71 (4.27, 17.25)	13.07 (11.83, 14.42)	
BMI (kg/m2)	29.10 (28.94,29.26)	26.12 (25.94, 26.30)	28.70 (28.49, 28.90)	30.28 (30.04, 30.52)	31.38 (31.17, 31.59)	<0.001
HTN (%)	38.20 (37.15, 39.25)	32.90 (31.30, 34.54)	36.35 (34.76, 37.96)	40.33 (38.70, 41.98)	43.34 (41.78, 44.93)	<0.001
DM (%)	14.38 (13.77, 15.02)	11.47 (10.59, 12.40)	13.43 (12.48, 14.45)	14.90 (13.73, 16.15)	17.76 (16.69, 18.88)	<0.001
CVD (%)	8.73 (8.25, 9.22)	9.42 (8.55, 10.37)	8.75 (7.97, 9.60)	8.44 (7.65, 9.30)	8.28 (7.45, 9.20)	0.207
Depression (%)	7.69 (7.24, 8.17)	6.48 (5.77, 7.26)	7.16 (6.30, 8.12)	8.27 (7.46, 9.16)	8.89 (8.16, 9.68)	<0.001
HbA1c (%)	5.61 (5.60, 5.63)	5.44 (5.42, 5.47)	5.55 (5.53, 5.57)	5.64 (5.61, 5.67)	5.82 (5.79, 5.86)	<0.001
Cholesterol (mmol/L)	5.02 (5.00,5.05)	4.46 (4.43, 4.50)	4.77 (4.74, 4.80)	5.10 (5.07, 5.13)	5.77 (5.73, 5.80)	<0.001
HDL cholesterol (mmol/L)	1.38 (1.37, 1.39)	1.82 (1.80, 1.84)	1.45 (1.44, 1.46)	1.24 (1.24, 1.25)	1.01 (1.00,1.02)	<0.001
Antidepressants (%)	13.17 (12.52, 13.84)	12.65 (11.66, 13.70)	13.76 (12.67, 14.93)	13.25 (12.25, 14.33)	13.01 (11.83, 14.28)	0.477
Anxiolytics, sedatives, and hypnotics (%)	6.78 (6.33, 7.27)	7.29 (6.54, 8.12)	6.78 (6.07, 7.56)	6.31 (5.56, 7.15)	6.75 (5.93, 7.68)	0.339

For continuous variables: survey-weighted mean (95% CI), the *p*-value was by survey-weighted linear regression (svyglm). For categorical variables: survey-weighted percentage (95% CI), the *p*-value was by survey-weighted Chi-square test (svytable).

Abbreviations: DM, diabetes mellitus; CVD: cardiovascular disease; BMI, body mass index; HbA1c, glycosylated hemoglobin; HDL, high-density lipoprotein; HTN, hypertension; PIR, poverty-income ratio (ratio of family income to poverty threshold); SD, standard deviation

Association between AC and depression

In the fully adjusted model, we observed a linear relationship between AC and depression (Figure 2). The results of the weighted multivariate logistic regression analysis are presented in Table 2. AC was positively correlated with depression in the crude model (odds ratio [OR]= 1.08, 95% confidence interval [CI]:1.06-1.11, *P* <0.001). A significant association between AC and depression was detected in Models 1-3 after adjusting for confounders. In Model 3, all variables were adjusted; for every 1 unit increase in AC, the incidence of depression increased by 3% (OR = 1.03, 95% CI = 1.00–1.06, *P* = 0.039).

Table 2. Associations of the atherogenic coefficient with depression (n = 32,502), Weighted.

For peer review only

	Crude Modela		Model 1b		Model 2c		Model 3d	
	**OR (95% CI)**	***p*-value**	**OR (95% CI)**	***p*-value**	**OR (95% CI)**	***p*-value**	**OR (95% CI)**	***p*-value**
Per 1 increase	1.08 (1.06,1.11)	<0.001	1.13 (1.10,1.16)	<0.001	1.07 (1.04,1.10)	<0.001	1.03 (1.00,1.06)	0.039
Quartiles								
Q1 (AC: < 1.9310)	Reference [1]		Reference [1]		Reference [1]		Reference [1]	
Q2 (AC: 1.9310 to < 2.6695)	1.11 (0.96,1.30)	0.166	1.18 (1.01,1.38)	0.040	1.07 (0.91,1.25)	0.431	1.04 (0.89,1.22)	0.589
Q3 (AC: 2.6695 to < 3.6430)	1.30 (1.11,1.53)	0.001	1.48 (1.26,1.74)	<0.001	1.24 (1.07,1.45)	0.006	1.18 (1.02,1.38)	0.034
Q4 (AC: ≥ 3.6430)	1.41 (1.22,1.62)	<0.001	1.75 (1.50,2.03)	<0.001	1.32 (1.14,1.54)	<0.001	1.15 (0.99,1.33)	0.074
p for trend	<0.001		<0.001		<0.001		0.040	

Abbreviations: AC, atherogenic coefficient.

aModel 1: Adjusted for age, sex, and race/ethnicity.

bModel 2: Adjusted for the variables in Model 1 plus body mass index, poverty-income ratio, educational level, and marital status.

cModel 3: Adjusted for the variables in Model 2 plus hypertension, diabetes mellitus, alcohol intake, smoking status, physical activity, and glycosylated hemoglobin.

After adjusting for age, sex, race/ethnicity, BMI, PIR, educational level, marital status, HTN, DM, alcohol intake, smoking status, physical activity, and glycosylated hemoglobin compared with participants in the first quartile (AC< 1.9310), the second group (1.9310 to < 2.6695, OR = 1.04, 95% CI = 0.89– 1.22, *P* = 0.589), the third group (2.6695 to < 3.6430, OR = 1.18, 95% CI = 1.02– 1.38, *P* = 0.034), and the fourth group (≥ 3.6430, OR = 1.15, 95% CI = 0.99– 1.33, *P* = 0.074) had an increased prevalence of depression (*P* for trend was significant in all the models).

Furthermore, regarding the interaction between sex and the relationship between AC and depression, the relationship was more significant in females (OR = 1.07, 95% CI = 1.02–1.12, *P* for interaction = 0.027) (Table 3).

Table 3. Subgroup analysis of the effect of the atherogenic coefficient on depression (n = 32502), Weight.

Subgroup	Number of	OR (95% CI)	*P* for

For peer review only - http://bmjopen.bmj.com/site/about/guidelines.xhtml

	participants		interaction
Sex, n (%)			0.027
Male	15954	1.05 (1.01, 1.10)	
Female	16548	1.07 (1.02, 1.12)	
Age, n (%)			0.375
20 – < 40	10857	1.07 (1.02, 1.12)	
40 – < 60	10632	1.04 (0.99, 1.09)	
≥ 60	11013	1.04 (0.97, 1.11)	
Race/ethnicity, n (%)			0.196
Non-Hispanic White	14112	1.03 (0.99, 1.08)	
Non-Hispanic Black	6713	1.06 (0.98, 1.15)	
Mexican American	5174	1.05 (0.97, 1.14)	
Other Hispanic	3109	1.12 (1.06, 1.19)	
Other race/multiple races	3394	1.10 (0.96, 1.26)	
BMI, kg/m2, mean (SD)			0.212
Low	10729	1.08 (1.00, 1.17)	
Middle	10733	1.06 (1.01, 1.12)	
High	10743	1.03 (0.99, 1.08)	
Hypertension, n (%)			0.949
Yes	13940	1.04 (1.00, 1.08)	
No	18562	1.06 (1.01, 1.12)	
DM, n (%)			0.670
Yes	6152	1.03 (0.98, 1.08)	
No	25761	1.07 (1.03, 1.11)	

Abbreviations: BMI, body mass index (calculated as weight, in kilograms, divided by the square of height, in meters); DM, diabetes mellitus; HTN, hypertension

DISCUSSION

This cross-sectional study showed an association between AC and depression in adults in the United States. After adjusting for covariates, a positive linear relationship was found between AC and depression.

The details of the mechanism explaining the relationship between AC and depression need to be further explored, and there may be several possible explanations. Lipids and the immune system interact with one another and have a regulatory effect on each other. Dysregulated inflammation promotes susceptibility to depression [23]. Studies have shown that inflammatory cytokines produced in the periphery enter the cells of the central nervous system and can affect neurotransmitters and neural circuits, producing behavioral symptoms of depression

For peer review only - http://bmjopen.bmj.com/site/about/guidelines.xhtml

[24]. When T lymphocytes are activated, they not only participate in immune inflammation but also directly contribute to the development of depression when functionally impaired [25,26]. Lipid peroxidation and oxidation-specific epitopes are formed, and the levels of antioxidants such as glutathione, glutathione peroxidase, and coenzyme Q10 are reduced, resulting in or aggravating oxidative stress [27,28].

When lipids are abnormal, the inflammatory, oxidative, and nitrosative (IO&NS) pathway is further activated [29]. At this time, the increase in binding globin and high-sensitivity C-reactive protein (CRP) is accompanied by a significant increase in interleukin-6 (IL-6), tumor necrosis factor-α, interferon-γ, other pro-inflammatory cytokines, and immune inflammation [30]. These factors lead to defects in serotonin and melatonin through the kynurenine pathway, often considered one of the main causes of depression [31]. Activation of the IO&NS pathway leads to mitochondrial and subsequent cellular dysfunction [32]. Previous studies have linked mitochondrial dysfunction in various brain regions to depression [33].

Statins have also been shown to have antidepressant effects when co-prescribed with antidepressants [34]. Lowering the AC index of patients with mood disorders improves CVD outcomes [35]. CVD is a heart and blood vessel disease characterized by myocardial infarction, angina pectoris, heart failure, heart attack, and stroke [36]. Longer exposure to depression is associated with significantly increased CVD risk [37]. Factors contributing to the link between depression and cardiac outcome may include alterations in the autonomic nervous system, platelet receptors and function, coagulopathic factors, pro-inflammatory cytokines, endothelial function, neurohormonal factors, and genetic linkages [38]. At the same time, patient compliance with antidepressant treatment is relatively poor [39]. Atherosclerosis is a chronic vascular inflammatory disease associated with oxidative stress and endothelial dysfunction [40]. Atherosclerosis is the underlying cause of CVD, and AC is a major indicator of atherosclerosis [41]. Our results suggest that AC may play a role in depression. AC may indicate the relationship between CVD and depression and a potential target and marker for treating depression or depression combined with CVD. The relevant mechanisms remain to be explored further.

For peer review only - http://bmjopen.bmj.com/site/about/guidelines.xhtml

Our study found increased odds of depression with increased AC in adults, demonstrating that controlling AC may be beneficial for preventing depression. Sex may affect this relationship. In the subgroup analysis (Table 3), we found a stronger relationship between AC and depression in females. The synergistic effect of estrogen on cognitive and emotional functions may underlie the association between ovarian hormone fluctuations and depression in females [42]. The induction of indoleamine 2, 3-dioxygenase, and deleterious effects of tryptophan catabolizing metabolites (TRYCATs) play a role in the pathophysiology of depression. Activation of IDO decreases plasma tryptophan levels and increases TRYCAT synthesis in depressed individuals. Females showed more IDO activation and TRYCAT production after immune challenge than males [43]. Therefore, this sex difference in immune dysregulation may contribute to higher levels of anxiety and depression experienced by females.

This study has some limitations. First, this was a cross-sectional study; therefore, we could not determine a causal relationship between AC and depression. Second, the PHQ-9 is a proven, simple, and effective tool for identifying the severity of depressive symptoms, but it is not a diagnostic tool for MDD. Third, the relationship we studied may have been influenced by other confounding factors, which we have not adjusted. Fourth, the differences in demographics and population characteristics in the United States may limit the generalizability of the findings to other countries or regions.

CONCLUSIONS

Our research shows that higher AC levels in American adults are positively related to a higher prevalence of depression. Further studies are required to explore the underlying mechanisms and potential benefits of controlling AC levels in patients with depression.

Funding: This work was supported by the Shandong Traditional Chinese Medicine Science and Technology Development Project under Grant 2019-0178.

For peer review only - http://bmjopen.bmj.com/site/about/guidelines.xhtml

Competing Interests: This research received no specific grant from any funding agency in the public, commercial, or not-for-profit sectors.

Author Contributions: Conceptualization, L Zhang, J Yin; methodology, L Zhang; software, J Yin; validation, H Sun, Y Liu and J Yang; formal analysis, L Zhang, J Yin, and H Sun; investigation, L Zhang; resources, L Zhang; data curation, H Sun; writing—original draft preparation, L Zhang; writing—review and editing, Y Liu and J Yang; visualization, J Yin; supervision, Y Liu and J Yang; project administration, L Zhang. All authors have read and agreed to the published version of the manuscript.

Acknowledgments: Thanks to the acupuncture-medicine team. Thanks to Prof. Liu and Prof. Yang for their careful teaching. Thanks to our colleagues for their help. Thanks to all who contributed to the manuscript.

Data Sharing Statement: The datasets generated and/or analyzed during the current study are available in the NHANES repository [https://www.cdc.gov/nchs/nhanes/].

For peer review only

References

[1]McCarron RM, Shapiro B, Rawles J, et al. Depression. Ann Intern Med. 2021;174:ITC65–ITC80.

[2]Malhi GS, Mann JJ. Depression. Lancet. 2018;392:2299–2312.

[3]Stringaris A. Editorial: what is depression? J Child Psychol Psychiatry. 2017;58:1287–1289.

[4]Reeder N, Tolar-Peterson T, Bailey RH, et al. Food Insecurity and Depression among US Adults: NHANES 2005-2016. Nutrients. 2022;14:3081.

[5]Meng R, Yu C, Liu N, et al. Association of Depression With All-Cause and Cardiovascular Disease Mortality Among Adults in China. JAMA Netw Open. 2020;3:e1921043.

[6]Hao Y, Ge H, Sun M, et al. Selecting an appropriate animal model of depression. Int J Mol Sci. 2019;20:4827.

[7]Edinoff AN, Akuly HA, Hanna TA, st al. Selective Serotonin Reuptake Inhibitors and Adverse Effects: A Narrative Review. Neurol Int. 2021;13:387-401.

[8]Andrews B, Klein B, Van Nguyen H, et al. Efficacy of a Digital Mental Health Biopsychosocial Transdiagnostic Intervention With or Without Therapist

For peer review only - http://bmjopen.bmj.com/site/about/guidelines.xhtml

Assistance for Adults With Anxiety and Depression: Adaptive Randomized Controlled Trial. J Med Internet Res. 2023;25:e45135.
[9]Milaneschi Y, Simmons WK, van Rossum EFC, et al. Depression and obesity: evidence of shared biological mechanisms. Mol Psychiatry. 2019;24:18–33.
[10]Able AA, Richard AJ, Stephens JM. TNFα Effects on Adipocytes Are Influenced by the Presence of Lysine Methyltransferases, G9a (EHMT2) and GLP (EHMT1). Biology (Basel). 2023;12:674.
[11]Otte C, Gold SM, Penninx BW, et al. Major depressive disorder. Nat Rev Dis Primers. 2016;2:16065.
[12]Beverly JK, Budoff MJ. Atherosclerosis: pathophysiology of insulin resistance, hyperglycemia, hyperlipidemia, and inflammation. J Diabetes. 2020;12:102–104.
[13]Mahdavi-Roshan M, Shoaibinobarian N, Noormohammadi M, et al. Inflammatory Markers and Atherogenic Coefficient: Early Markers of Metabolic Syndrome. Int J Endocrinol Metab. 2022;20:e127445.
[14]Nunes SO, Piccoli de Melo LG, Pizzo de Castro MR, et al. Atherogenic index of plasma and atherogenic coefficient are increased in major depression and bipolar disorder, especially when comorbid with tobacco use disorder. J Affect Disord. 2015;172:55–62.
[15]Tao SH, Ren XQ, Zhang LJ, et al. Association between common cardiovascular drugs and depression. Chin Med J (Engl). 2021;134:2656–2665.
[16]Walker AJ, Kim Y, Borissiouk I, et al. Statins: neurobiological underpinnings and mechanisms in mood disorders. Neurosci Biobehav Rev. 2021;128:693–708.
[17]Fain JA. NHANES. Diabetes Educ. 2017;43:151.
[18]Patel JS, Oh Y, Rand KL, et al. Measurement invariance of the patient health questionnaire-9 (PHQ-9) depression screener in U.S. adults across sex, race/ethnicity, and education level: NHANES 2005-2016. Depress Anxiety. 2019;36:813-823.
[19]Levis B, Benedetti A, Thombs BD, et al. Accuracy of Patient Health Questionnaire-9 (PHQ-9) for screening to detect major depression: individual participant data meta-analysis. BMJ. 2019;365:l1476.
[20]Brooks JM, Titus AJ, Bruce ML, et al. Depression and handgrip strength among U.S. adults aged 60 years and older from NHANES 2011-2014. J Nutr Health Aging. 2018;22:938–943.

For peer review only - http://bmjopen.bmj.com/site/about/guidelines.xhtml

[21]Levis B, Benedetti A, Thombs BD; DEPRESsion Screening Data (DEPRESSD) Collaboration. Accuracy of Patient Health Questionnaire-9 (PHQ-9) for screening to detect major depression: individual participant data meta-analysis. BMJ. 2019;365:l1476.

[22]Stekhoven DJ, Bühlmann P. MissForest—non-parametric missing value imputation for mixed-type data. Bioinformatics. 2012;28:112–118.

[23]Beurel E, Toups M, Nemeroff CB. The bidirectional relationship of depression and inflammation: double trouble. Neuron. 2020;107:234–256.

[24]Capuron L, Lasselin J, Castanon N. Role of adiposity-driven inflammation in depressive morbidity. Neuropsychopharmacology. 2017;42:115–128.

[25]Haapakoski R, Ebmeier KP, Alenius H, et al. Innate and adaptive immunity in the development of depression: an update on current knowledge and technological advances. Prog Neuropsychopharmacol Biol Psychiatry. 2016;66:63–72.

[26]Miller AH. Depression and immunity: a role for T cells? Brain Behav Immun. 2010;24:1–8.

[27]Bartolini Gritti B, Binder CJ. Oxidation-specific epitopes are major targets of innate immunity in atherothrombosis. Hamostaseologie. 2016;36:89–96.

[28]Maes M, Mihaylova I, Kubera M, et al. Increased plasma peroxides and serum oxidized low density lipoprotein antibodies in major depression: markers that further explain the higher incidence of neurodegeneration and coronary artery disease. J Affect Disord. 2010;125:287–94.

[29]Morris G, Puri BK, Walker AJ, et al. Shared pathways for neuroprogression and somatoprogression in neuropsychiatric disorders. Neurosci Biobehav Rev. 2019;107:862–882.

[30]Maes M, Ruckoanich P, Chang YS, et al. Multiple aberrations in shared inflammatory and oxidative & nitrosative stress (IO&NS) pathways explain the co-association of depression and cardiovascular disorder (CVD), and the increased risk for CVD and due mortality in depressed patients. Prog Neuropsychopharmacol Biol Psychiatry. 2011;35:769–783.

[31]Gałecki P, Talarowska M. Inflammatory theory of depression. Psychiatr Pol. 2018;52:437–447.

[32]Mezzaroba L, Alfieri DF, Colado Simão AN, et al. The role of zinc, copper, manganese and iron in neurodegenerative diseases. Neurotoxicology. 2019;74:230–241.

For peer review only

[33]Bansal Y, Kuhad A. Mitochondrial dysfunction in depression. Curr Neuropharmacol. 2016;4:610–618.

[34]Singh D, Lippmann S. Can statins diminish depression? Prim Care Companion CNS Disord. 2018;20:17br02169.

[35]Zhang Y, Chen Y, Ma L. Depression and cardiovascular disease in elderly: current understanding. J Clin Neurosci. 2018;47:1–5.

[36]Xu C, Liang J, Xu S, et al. Increased serum levels of aldehydes are associated with cardiovascular disease and cardiovascular risk factors in adults. J Hazard Mater. 2020;400:123134.

[37]Zhang Y, Li X, Chan VKY, et al. Depression duration and risk of incident cardiovascular disease: A population-based six-year cohort study. J Affect Disord. 2022;305:188-195.

[38]de Jonge P, Rosmalen JG, Kema IP, et al. Psychophysiological biomarkers explaining the association between depression and prognosis in coronary artery patients: a critical review of the literature. Neurosci Biobehav Rev. 2010;35:84–90.

[39]Shrestha Manandhar J , Shrestha R , Basnet N , et al. Study of Adherence Pattern of Antidepressants in Patients with Depression. Kathmandu Univ Med J (KUMJ). 2017;15:3-9.

[40]Marchio P, Guerra-Ojeda S, Vila JM, et al. Targeting early atherosclerosis: a focus on oxidative stress and inflammation. Oxid Med Cell Longev. 2019;2019:8563845.

[41]Nitsa A, Toutouza M, Machairas N, et al. Vitamin D in cardiovascular disease. In Vivo. 2018;32:977–981.

[42]Albert KM, Newhouse PA. Estrogen, stress, and depression: cognitive and biological interactions. Annu Rev Clin Psychol. 2019;15:399–423.

[43]Maes M, Leonard BE, Myint AM, et al. The new '5-HT' hypothesis of depression: cell-mediated immune activation induces indoleamine 2,3-dioxygenase, which leads to lower plasma tryptophan and an increased synthesis of detrimental tryptophan catabolites (TRYCATs), both of which contribute to the onset of depression. Prog Neuropsychopharmacol Biol Psychiatry. 2011;35:702–721.

FIGURE LEGENDS

For peer review only - http://bmjopen.bmj.com/site/about/guidelines.xhtml

Figure 1. Flowchart for inclusion of study participants.

Figure 2. Association between AC and depression in US adults (n=4759). The black vertical line on the horizontal axis represents the AC distribution, the red line represents the best fit, and the difference between the dashed lines represents the 95% confidence interval. The data was adjusted for age, sex, race/ethnicity, body mass index, poverty-income ratio, educational level, marital status, hypertension, diabetes mellitus, alcohol intake, smoking status, physical activity, and glycosylated hemoglobin.

For peer review only

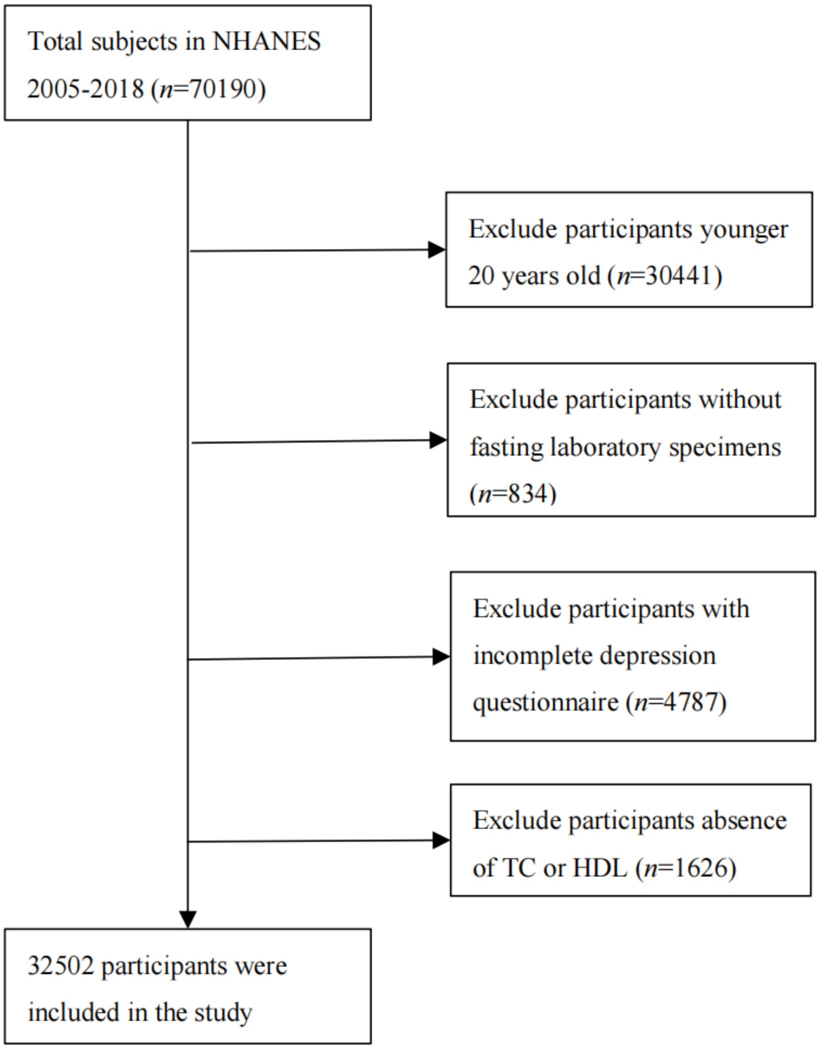

Figure 1. Flowchart for inclusion of study participants.

Flowchart for inclusion of study participants.

114x140mm (200 x 200 DPI)

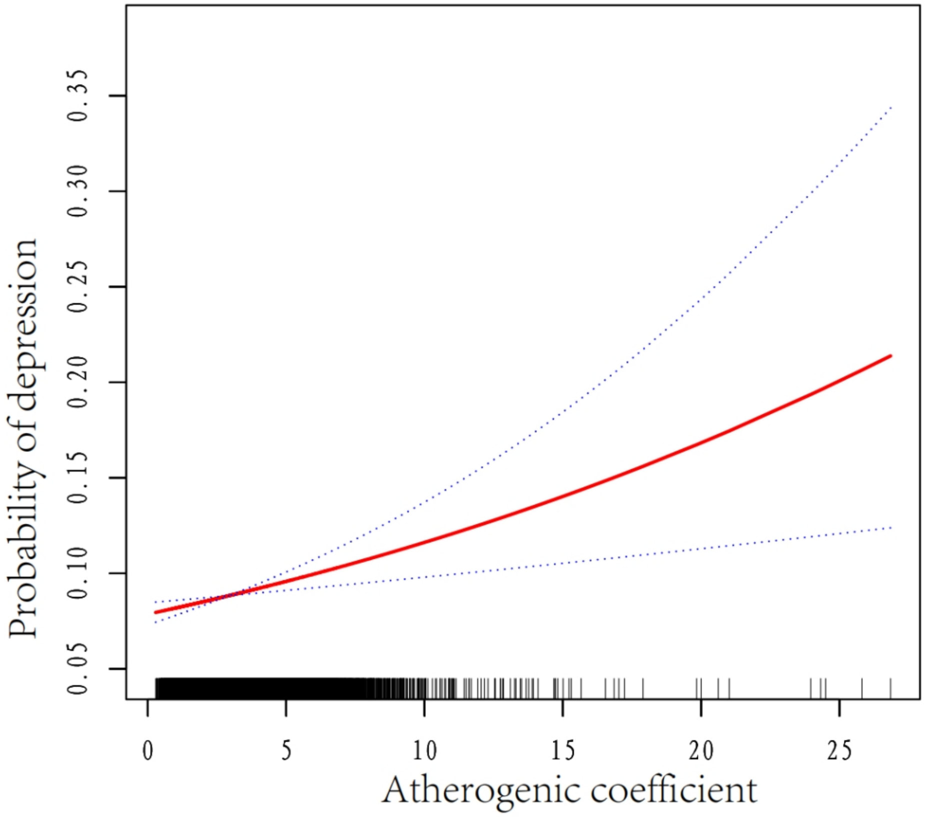

Association between AC and depression in US adults (n=4759). The black vertical line on the horizontal axis represents the caffeine distribution, the red line represents the best fit, and the difference between the dashed lines represents the 95% confidence interval. The data was adjusted for age, sex, race/ethnicity, body mass index, poverty-income ratio, educational level, marital status, hypertension, diabetes mellitus, alcohol intake, smoking status, physical activity, and glycosylated hemoglobin.

152x152mm (200 x 200 DPI)

Table S1. Missing covariates of study participants (n = 32502)

Variable	Number of patients (% missing)
Age	0 (0%)
Sex	0 (0%)
Race/ethnicity	0 (0%)
Educational level	23 (0.07%)
Marital status	16 (0.04%)
Poverty-income ratio	2767 (8.51%)
Body mass index	297 (0.91%)
Alcohol intake	4661 (14.34%)
Smoking status	17 (0.05%)
Physical activity	4102 (12.62%)
Hypertension	0 (0%)
Diabetes mellitus	589 (1.81%)
Glycosylated hemoglobin	58 (0.18%)

For peer review only

STROBE 2007 (v4) Statement—Checklist of items that should be included in reports of *cross-sectional studies*

**Section/Topic**	**Item #**	**Recommendation**	**Reported on page #**
**Title and abstract**	1	(*a*) Indicate the study's design with a commonly used term in the title or the abstract	2
		(*b*) Provide in the abstract an informative and balanced summary of what was done and what was found	2
**Introduction**			
Background/rationale	2	Explain the scientific background and rationale for the investigation being reported	3
Objectives	3	State specific objectives, including any prespecified hypotheses	4
**Methods**			
Study design	4	Present key elements of study design early in the paper	
Setting	5	Describe the setting, locations, and relevant dates, including periods of recruitment, exposure, follow-up, and data collection	
Participants	6	(*a*) Give the eligibility criteria, and the sources and methods of selection of participants	4
Variables	7	Clearly define all outcomes, exposures, predictors, potential confounders, and effect modifiers. Give diagnostic criteria, if applicable	5-6
Data sources/ measurement	8*	For each variable of interest, give sources of data and details of methods of assessment (measurement). Describe comparability of assessment methods if there is more than one group	5-6
Bias	9	Describe any efforts to address potential sources of bias	*6-7*
Study size	10	Explain how the study size was arrived at	4
Quantitative variables	11	Explain how quantitative variables were handled in the analyses. If applicable, describe which groupings were chosen and why	5-6
Statistical methods	12	(*a*) Describe all statistical methods, including those used to control for confounding	6-7
		(*b*) Describe any methods used to examine subgroups and interactions	5
		(*c*) Explain how missing data were addressed	7
		(*d*) If applicable, describe analytical methods taking account of sampling strategy	
		(*e*) Describe any sensitivity analyses	
**Results**			

For peer review only

Participants	13*	(a) Report numbers of individuals at each stage of study—eg numbers potentially eligible, examined for eligibility, confirmed eligible, included in the study, completing follow-up, and analysed	7
		(b) Give reasons for non-participation at each stage	
		(c) Consider use of a flow diagram	7
Descriptive data	14*	(a) Give characteristics of study participants (eg demographic, clinical, social) and information on exposures and potential confounders	4
		(b) Indicate number of participants with missing data for each variable of interest	
Outcome data	15*	Report numbers of outcome events or summary measures	7
Main results	16	(*a*) Give unadjusted estimates and, if applicable, confounder-adjusted estimates and their precision (eg, 95% confidence interval). Make clear which confounders were adjusted for and why they were included	5-6
		(*b*) Report category boundaries when continuous variables were categorized	
		(*c*) If relevant, consider translating estimates of relative risk into absolute risk for a meaningful time period	
Other analyses	17	Report other analyses done—eg analyses of subgroups and interactions, and sensitivity analyses	12
**Discussion**			
Key results	18	Summarise key results with reference to study objectives	13-14
Limitations	19	Discuss limitations of the study, taking into account sources of potential bias or imprecision. Discuss both direction and magnitude of any potential bias	15
Interpretation	20	Give a cautious overall interpretation of results considering objectives, limitations, multiplicity of analyses, results from similar studies, and other relevant evidence	
Generalisability	21	Discuss the generalisability (external validity) of the study results	15
**Other information**			
Funding	22	Give the source of funding and the role of the funders for the present study and, if applicable, for the original study on which the present article is based	15

*Give information separately for cases and controls in case-control studies and, if applicable, for exposed and unexposed groups in cohort and cross-sectional studies.

Note: An Explanation and Elaboration article discusses each checklist item and gives methodological background and published examples of transparent reporting. The STROBE checklist is best used in conjunction with this article (freely available on the Web sites of PLoS Medicine at http://www.plosmedicine.org/, Annals of Internal Medicine at http://www.annals.org/, and Epidemiology at http://www.epidem.com/). Information on the STROBE Initiative is available at www.strobe-statement.org.

For peer review only

BMJ Open

Association between atherogenic coefficient and depression in US adults: a cross-sectional study with data from National Health and Nutrition Examination Survey 2005-2018

Journal:	*BMJ Open*
Manuscript ID	bmjopen-2023-074001.R2
Article Type:	Original research
Date Submitted by the Author:	03-Oct-2023
Complete List of Authors:	Zhang, Lu; Shandong University of Traditional Chinese Medicine<br>Yin, Jiahui; Shandong University of Traditional Chinese Medicine<br>Sun, Haiyang; Guangzhou University of Chinese Medicine<br>Yang, Jiguo; Shandong University of Traditional Chinese Medicine<br>Liu, Yuanxiang; Affiliated Hospital of Shandong University of Traditional Chinese Medicine
**Primary Subject Heading**:	Mental health
Secondary Subject Heading:	Health services research
Keywords:	Risk Factors, Adult psychiatry < PSYCHIATRY, Depression & mood disorders < PSYCHIATRY, Lipid disorders < DIABETES & ENDOCRINOLOGY

SCHOLARONE™
Manuscripts

I, the Submitting Author has the right to grant and does grant on behalf of all authors of the Work (as defined in the below author licence), an exclusive licence and/or a non-exclusive licence for contributions from authors who are: i) UK Crown employees; ii) where BMJ has agreed a CC-BY licence shall apply, and/or iii) in accordance with the terms applicable for US Federal Government officers or employees acting as part of their official duties; on a worldwide, perpetual, irrevocable, royalty-free basis to BMJ Publishing Group Ltd ("BMJ") its licensees and where the relevant Journal is co-owned by BMJ to the co-owners of the Journal, to publish the Work in this journal and any other BMJ products and to exploit all rights, as set out in our licence.

The Submitting Author accepts and understands that any supply made under these terms is made by BMJ to the Submitting Author unless you are acting as an employee on behalf of your employer or a postgraduate student of an affiliated institution which is paying any applicable article publishing charge ("APC") for Open Access articles. Where the Submitting Author wishes to make the Work available on an Open Access basis (and intends to pay the relevant APC), the terms of reuse of such Open Access shall be governed by a Creative Commons licence – details of these licences and which Creative Commons licence will apply to this Work are set out in our licence referred to above.

Other than as permitted in any relevant BMJ Author's Self Archiving Policies, I confirm this Work has not been accepted for publication elsewhere, is not being considered for publication elsewhere and does not duplicate material already published. I confirm all authors consent to publication of this Work and authorise the granting of this licence.

For peer review only

Association between atherogenic coefficient and depression in US adults: a cross-sectional study with data from National Health and Nutrition Examination Survey 2005-2018

Lu Zhang[a1], Jiahui Yin[b1], Haiyang Sun[c1], Jiguo Yang[d*], Yuanxiang Liu[e*]

[a]*Shandong University of Traditional Chinese Medicine, Jinan, China.*

[b]*Shandong University of Traditional Chinese Medicine, Jinan, China.*

[c]*Guangzhou University of Chinese Medicine, Guangzhou, China.*

[d]*Shandong University of Traditional Chinese Medicine, Jinan, China.*

[e]*Affiliated Hospital of Shandong University of Traditional Chinese Medicine, Jinan, China.*

* Correspondence: Yuanxiang Liu: lyxlwtg001@163.com;

Address: No.16369 Jingshi Road, Lixia District, Jinan, China

[1]These authors contributed equally to this work

For peer review only

For peer review only - http://bmjopen.bmj.com/site/about/guidelines.xhtml

ABSTRACT

Objective: The pathogenesis of depression is related to immune inflammatory response. AC is an important indicator of lipid abnormalities, which can lead to immune inflammatory responses. However, no study has investigated the relationship between AC and depression in adult Americans. Therefore, we investigated this relationship.

Design: This study used a cross-sectional design.

Setting: The National Health and Nutrition Examination Survey (2005-2018) data were used for this study.

Participants: A total of 32502 participants aged 20 years or older who had complete information for AC and depression were included in this study.

Primary and secondary outcome measures: Depressive symptoms were assessed using the nine-item version of the Patient Health Questionnaire (PHQ-9), with a cutoff point of 9/10 indicating likely depression cases. Weighted logistic regression analyses and the smooth curve fittings were performed to explore the association between AC and depression.

Results: After adjusting for potential confounders, a single unit increase in AC was associated with a 3% increase in the prevalence of depression (hazard ratio =1.03, 95% confidence interval =1.00-1.06, *P* = 0.039). The relationship between AC and depression was more obvious in females.

Conclusions: The AC is positively associated with depression.

Strengths and Limitations:

- The quality and scale of the National Health and Nutrition Examination Survey database ensured our results' statistical power and reliability.
- A wide range of sociodemographic, lifestyle, and physical health covariates were adjusted to reduce residual confounding.
- Its cross-sectional design limited this study, and no causal relationships could be determined.
- The PHQ-9 is a proven, simple, and effective tool for identifying the severity of depressive symptoms, but it is not a diagnostic tool for major depressive disorder (MDD).

For peer review only - http://bmjopen.bmj.com/site/about/guidelines.xhtml

INTRODUCTION

Depression is a clinically common emotional state characterized by persistent sadness or inability to experience pleasure, accompanied by deficits in daily functioning [1]. In 2008, the World Health Organization (WHO) ranked major depression as the third leading cause of the global disease burden and projected that it would be the number one cause by 2030 [2]. More than 300 million people worldwide suffer from major depressive disorder (MDD) [3], affecting about 8% of adults in the US [4]. Depression can cause various adverse events, seriously endangering lives and global health [5,6]. Current antidepressant treatments are effective, but there are many side effects; for example, antidepressants may increase suicidal thoughts in some people [7]. Evidence supports screening for depression and providing early intervention [8]. Therefore, it is necessary to explore the factors related to depression.

Abnormal lipid metabolism leads to many pathological changes. Firstly, activation of the pro-inflammatory response leads to a decrease in high-density lipoprotein (HDL) and phospholipids and a compensatory increase in phospholipid-rich low-density lipoprotein (LDL), which in turn slows total cholesterol (TC) metabolism and affects neurotransmitters and neural circuits [9]. However, cytokine signaling in adipose tissue, particularly tumor necrosis factor (TNF), promotes metabolic dysregulation [10]. In addition, some studies have shown that changes in circulating lipid concentrations may be associated with depression [11]. Abnormal lipids are involved in the formation of atherosclerosis. The pathogenesis of atherosclerosis is based on the lipid theory, and the explanation is related to excess cholesterol being the sole cause of lipid deposition in the arterial wall [12]. Atherosclerosis can cause cardiovascular disease (CVD), stroke, etc., often co-morbidities with depression.

The atherogenic coefficient (AC) is an important index for assessing the degree of atherosclerosis, calculated as (TC – HDL)/HDL [13]. Nunes et al. found that AC was elevated in patients with MDD and bipolar disorder [14]. AC and depression can be controlled using statins and other cardiovascular drugs [15,16].

For peer review only - http://bmjopen.bmj.com/site/about/guidelines.xhtml

Exploring the role of AC in depression may be beneficial for treating depression and its complications. Therefore, we used data from the National Health and Nutrition Examination Survey (NHANES) database to explore the association of AC with depression in adults.

METHODS

Study design and participants

Data of the participants in this study were obtained from the NHANES database, a major program conducted by the Centers for Disease Control and Prevention (CDC) to assess the health and nutritional status of 5,000 adults and children in the US annually [17]. The NHANES database contains demographic, dietary, examination, laboratory, and questionnaire data. The National Center for Health Statistics (NCHS) Research Ethics Review Board (ERB) authorized the NHANES study protocols. Further information regarding the NHANES data can be obtained from its official website (http://www.cdc.gov/nchs/nhanes.htm).

Participants in our study were screened according to the following inclusion criteria: 1) aged 20 years or above and 2) participation in laboratory tests on an empty stomach. The exclusion criteria were: 1) incomplete Patient Health Questionnaire-9 (PHQ-9) and 2) no data on TC or HDL cholesterol levels.

Assessment of depression

The PHQ-9 is used to assess depression. The PHQ-9 contains nine items that capture the frequency of depressive symptoms: appetite problems, fatigue, sleep difficulties, psychomotor retardation or agitation, concentration problems, lack of interest, depressed mood, feelings of worthlessness, and suicidal ideation. It is now widely accepted as an accurate and reliable method for screening depression [18,19]. Each question is scored from '0' (not at all) to '3' (nearly every day), with a total score of 0–27, where a score ≥10 is considered clinically relevant depression (CRD) [20]. PHQ-9 sensitivity compared with semi-structured diagnostic interviews was greater than previous conventional meta-analyses that combined reference standards. A 10- or

For peer review only - http://bmjopen.bmj.com/site/about/guidelines.xhtml

above cutoff score maximized the overall sensitivity and specificity for subgroups [21].

Assessment of AC

Fasting blood was drawn from individuals aged ≥ 20 years, and the blood samples were processed, stored, and shipped to the Johns Hopkins University Lipoprotein Assay Laboratory at the Ambulator-Testing Center laboratory. HDL cholesterol levels were measured directly in the serum. The apolipoprotein B (apo B)-containing lipoproteins in the specimen were reacted with a blocking reagent that rendered them non-reactive with the enzymatic cholesterol reagent under the assay conditions. Reagents were purchased from Roche/Boehringer-Mannheim Diagnostics (Mannheim, Germany). The method uses sulfated alpha-cyclodextrin in the presence of Mg^{+2}, which forms complexes with apoB-containing lipoproteins and polyethylene glycol-coupled cholesteryl esterase and cholesterol oxidase for HDL cholesterol measurement. HDL cholesterol data collected from participants in 2005-2006 were adjusted using the following equation: corrected HDL = (Solomon Park assigned HDL value) × (participant HDL). TC was measured enzymatically in the serum or plasma in a series of coupled reactions that hydrolyze cholesteryl esters and oxidize the 3-OH cholesterol group. All the information can be obtained from https://wwwn.cdc.gov/Nchs/Nhanes/.

Assessment of covariates

Covariates in this study, including body mass index (BMI), alcohol intake, and glycosylated hemoglobin (HbA1c), were used as continuous variables. BMI was measured as weight (kg) divided by height (m) squared with <25.0 kg/m2 indicating normal, 25.0 to <30.0 kg/m2 indicating overweight, ≥30.0 kg/m2 indicating obesity. Alcohol intake was determined by extracting the mean alcohol intake from the first and second dietary surveys, considering a single day's intake for participants who consumed alcohol at least once. Physical activity was self-reported by participants as either inactive, moderate, or vigorous. Categorical variables included age (20–40 years, 40–60 years, ≥60 years), sex (male or female), and race/ethnicity (non-Hispanic white, non-Hispanic black, Mexican American, other Hispanic, or other race/multiple

For peer review only - http://bmjopen.bmj.com/site/about/guidelines.xhtml

races). The poverty-income ratio (PIR) was defined as the ratio of family income to poverty threshold (<1 indicating an income below the poverty threshold and ≥1 indicating an income above the poverty threshold. The latter category was further classified into two groups: 1.00 to <2.00 and ≥2.00). Education level was categorized as high school not completed, high school completed, or high school graduate and some college or associated degrees pursued. Marital status was defined as married/living with a partner or widowed/divorced/separated/never married. Hypertension (HTN) (defined as systolic blood pressure ≥140 mmHg or diastolic blood pressure ≥90 mmHg) was determined using three blood pressure measurements at different times, an existing diagnosis, or evidence of an existing antihypertensive medication regimen. Diabetes mellitus (DM) was defined as either taking glucose-lowering therapies, HbA1c concentration of ≥6.5%, use of anti-diabetic medication, oral glucose tolerance test (OGTT) ≥11.1 mmol/L, fasting plasma glucose ≥7.0 mmol/L, or random blood glucose ≥11.1 mmol/L. Smoking status was categorized as non-smokers (smoked <100 cigarettes in a lifetime), former smoker (not currently smoking but have consumed ≥100 cigarettes previously), and current smoker (smoking at least ≥100 cigarettes every day or some days).

Statistical analysis

The main concern was whether AC is associated with depression after adjusting for other factors that may influence depression. Continuous variables are expressed as mean ± standard deviation, and categorical variables are expressed as percentages. The weighted $\chi 2$ test was used to compare categorical variables between groups, a one-way analysis of variance was used to compare normally distributed variables between groups, and the Kruskal-Wallis H test was used to compare variables with a skewed distribution between groups. Variance inflation factors were used to test multi-collinearity. Weighted multivariate logistic regression analysis evaluated the independent association between AC and depression. The participants were categorized into four groups based on AC: < 1.9310, 1.9310 to < 2.6695, 2.6695 to < 3.6430, and ≥ 3.6430. We used three levels of adjustment: Model 1 was adjusted for age, sex, and race/ethnicity; Model 2 was adjusted for the variables in Model 1 plus

For peer review only - http://bmjopen.bmj.com/site/about/guidelines.xhtml

BMI, PIR, educational level, and marital status; and Model 3 was adjusted for the variables in Model 2 plus HTN, DM, alcohol intake, smoking status, physical activity, and HbA1c. The imputation of missing data was conducted using the missForest R package. This random forest-based technique is highly computationally efficient for high-dimensional data of categorical and continuous predictors [22]. The missing values are presented in the table (Table S1).

All analyses were performed using R software (The R Foundation, Vienna, Austria) and Empower (X&Y Solutions, Boston, MA, USA). Statistical significance was defined as a two-sided *P*-value <0.05.

Patient and public involvement

None.

RESULTS

Participant characteristics

In this study, 32,502 participants were included (Figure 1). Table 1 shows the characteristics of the participants according to their AC. There were statistically significant differences in age, sex, educational level, race/ethnicity, marital status, PIR, alcohol intake, smoking status, physical activity, BMI, HTN, DM, HbA1c, TC, and HDL cholesterol between the different AC groups (*P* <0.05).

In addition to that, among those aged 20 years or older (n=30,441), 3,891 (9.79%) participants had missing AC values. The proportion of missing values for the different age groups is shown in Table S2. Fewer proportions of people between 40 and 69 years old had missing AC values compared to those under 40 and those over 70 years.

We conducted a threshold saturation effect analysis on the data, and the results suggested a linear correlation between AC and depression (log-likelihood ratio (LLR)=0.051). The results of the threshold saturation effect are displayed in Table S3. Covariance is generally indicated if the tolerance (Tol) is less than 0.1 or the variance inflation factor (VIF) is greater than 10. Therefore, our results can initially ignore the problem of multicollinearity (Table S4).

For peer review only

For peer review only - http://bmjopen.bmj.com/site/about/guidelines.xhtml

Participants in the lowest AC in Q1 (< 1.9310) were likely to be female, younger, more educated, married or cohabitating, non-Hispanic White, wealthier, less physically active, smoked less, consumed more alcohol, had no DM or HTN, higher HDL cholesterol levels, lower BMI, lower HbA1c levels, and TC levels.

In contrast, participants with the highest AC in Q4 (>3.6430) were likely to be male, middle-aged, more highly educated, non-Hispanic White, married or cohabitating, wealthier, consumed less alcohol, never smoked, inactive and obese, had HTN, lower HDL cholesterol levels, higher BMI, higher HbA1c and TC levels.

Table 1. Characteristics of the study population, using National Health and Nutrition Examination Survey data from 2005–2018 (N = 32,502), Weighted

Characteristic	Overall	Atherogenic coefficient quartiles†				*p*-value
		Q1 (< 1.9310)	Q2 (1.9310 to < 2.6695)	Q3 (2.6695 to < 3.6430)	Q4 (≥ 3.6430)	
Sex (%)						<0.001
Male	48.78 (48.21,49.35)	32.75 (31.33, 34.21)	42.40 (40.95, 43.87)	54.04 (52.56,55.52)	66.36 (65.00,67.70)	
Female	51.22 (50.65, 51.79)	67.25 (65.79, 68.67)	57.60 (56.13, 59.05)	45.96 (44.48, 47.44)	33.64 (32.30, 35.00)	
Age (%)						<0.001
20 to < 40	35.93 (34.76, 37.12)	40.78 (38.93, 42.66)	36.40 (34.73, 38.10)	33.15 (31.58, 34.76)	33.30 (31.69, 34.95)	
40 to <60	37.75 (36.83, 38.67)	29.54 (27.96, 31.18)	35.21 (33.59, 36.87)	40.86 (39.37,42.37)	45.58 (43.89, 47.29)	
≥60	26.32 (25.26.27.40)	29.67 (28.04, 31.36)	28.39 (26.74, 30.09)	25.99 (24.53, 27.50)	21.12 (19.78,22.52)	
Educational level (%)						<0.001
<High school	15.44 (14.36, 16.58)	12.40 (11.24, 13.65)	14.04 (12.70, 15.49)	16.18 (14.78, 17.67)	19.24 (17.90, 20.65)	
Completed high school	23.30 (22.35, 24.27)	20.49 (19.12, 21.93)	22.16 (20.82, 23.56)	24.87 (23.59,26.20)	25.75 (24.17, 27.40)	
>High school	61.26 (59.57, 62.92)	67.11 (65.08, 69.08)	63.80 (61.69, 65.86)	58.95 (56.99, 60.89)	55.02 (52.92, 57.09)	
Race/ethnicity (%)						<0.001
Non-Hispanic White	68.41 (65.86, 70.86)	69.02 (66.42, 71.51)	68.41 (65.87, 70.84)	68.23 (65.19, 71.72)	67.98 (65.01, 70.81)	
Non-Hispanic Black	10.50 (9.27,11.88)	13.76 (12.07, 15.64)	11.32 (9.98, 12.81)	9.65 (8.42, 11.03)	7.20 (6.22, 8.32)	
Mexican American	8.46 (7.22, 9.89)	6.06 (5.10, 7.18)	7.53 (6.34, 8.93)	9.50 (8.04, 11.20)	10.82 (9.06, 12.86)	
Other Hispanic	5.44 (4.65,6.35)	4.27 (3.54, 5.13)	5.22 (4.40, 6.18)	5.83 (4.87, 6.97)	6.47 (5.42, 7.70)	
Other races/multiple races	7.18 (6.50, 7.93)	6.89 (6.08, 7.81)	7.52 (6.63, 8.51)	6.79 (5.96, 7.73)	7.54 (6.67, 8.50)	
Marital status (%)						<0.001
Married/Living with a partner	64.04 (62.84, 65.22)	59.25 (57.29, 61.17)	62.92 (61.31, 64.54)	66.06 (64.48, 67.61)	68.04 (66.58,69.46)	
Widowed/Divorced/Sepa	35.96 (34.78, 37.16)	40.75 (38.83, 42.71)	37.08 (35.49, 38.69)	33.94 (32.39, 35.52)	31.96 (30.54, 33.42)	

For peer review only
For peer review only - http://bmjopen.bmj.com/site/about/guidelines.xhtml

rated/Never married						
PIR (%)						<0.001
< 1.00	13.58 (12.64, 14.57)	12.69 (11.51, 13.97)	13.36 (12.18, 14.64)	12.77 (11.70, 13.92)	15.51 (14.13, 17.00)	
1.00 to <2.00	20.31 (19.33, 21.32)	18.88 (17.52, 20.31)	19.48 (18.36, 20.65)	21.07 (19.63, 22.60)	21.83 (20.18, 23.57)	
≥2.00	66.12 (64.45, 67.74)	68.44 (66.44, 70.37)	67.16 (65.18, 69.08)	66.15 (64.30, 67.96)	62.66 (60.14, 65.12)	
Alcohol intake (g/day)	9.38 (8.90,9.87)	12.33 (11.43, 13.24)	8.91 (8.22, 9.61)	7.84 (7.19, 8.49)	8.43 (7.62, 9.25)	<0.001
Smoking status (%)						<0.001
Non-smoker	54.76 (53.63, 55.88)	58.23 (56.66, 59.78)	56.97 (55.26, 58.66)	54.94 (53.42, 56.45)	48.79 (47.25, 50.33)	
Former smoker	25.13 (24.28, 26.00)	24.55 (23.30, 25.85)	24.60 (23.23, 26.02)	26.12 (24.65, 27.66)	25.26 (24.00, 26.56)	
Current smoker	20.11 (19.24, 21.01)	17.22 (16.03, 18.48)	18.44 (17.19, 19.75)	18.94 (17.85, 20.08)	25.95 (24.45,27.51)	
Physical activity (%)						<0.001
Inactive	45.81 (44.18, 47.45)	39.32 (36.97, 41.72)	44.08 (41.77, 46.42)	47.93 (46.10, 49.77)	52.15 (50.11, 54.17)	
Moderate	28.01 (26.94, 29.11)	27.48 (25.78, 29.25)	28.01 (26.30, 29.78)	29.11 (27.52, 30.76)	27.43 (25.81, 29.11)	
Vigorous	8.05 (7.55, 8.59)	8.86 (7.84, 9.99)	8.74 (7.74, 9.85)	7.25 (6.40, 8.20)	7.36 (6.37, 8.48)	
Both moderate and vigorous	18.13 (116.98, 19.34)	24.34 (22.46, 26.33)	19.17 (17.54, 20.93)	15.71 (4.27, 17.25)	13.07 (11.83, 14.42)	
BMI (kg/m2)	29.10 (28.94,29.26)	26.12 (25.94, 26.30)	28.70 (28.49, 28.90)	30.28 (30.04, 30.52)	31.38 (31.17, 31.59)	<0.001
HTN (%)	38.20 (37.15, 39.25)	32.90 (31.30, 34.54)	36.35 (34.76, 37.96)	40.33 (38.70, 41.98)	43.34 (41.78, 44.93)	<0.001
DM (%)	14.38 (13.77, 15.02)	11.47 (10.59, 12.40)	13.43 (12.48, 14.45)	14.90 (13.73, 16.15)	17.76 (16.69, 18.88)	<0.001
CVD (%)	8.73 (8.25, 9.22)	9.42 (8.55, 10.37)	8.75 (7.97, 9.60)	8.44 (7.65, 9.30)	8.28 (7.45, 9.20)	0.207
Depression (%)	7.69 (7.24, 8.17)	6.48 (5.77, 7.26)	7.16 (6.30, 8.12)	8.27 (7.46, 9.16)	8.89 (8.16, 9.68)	<0.001
HbA1c (%)	5.61 (5.60, 5.63)	5.44 (5.42, 5.47)	5.55 (5.53, 5.57)	5.64 (5.61, 5.67)	5.82 (5.79, 5.86)	<0.001
TC(mmol/L)	5.02 (5.00,5.05)	4.46 (4.43, 4.50)	4.77 (4.74, 4.80)	5.10 (5.07, 5.13)	5.77 (5.73, 5.80)	<0.001
HDL cholesterol (mmol/L)	1.38 (1.37, 1.39)	1.82 (1.80, 1.84)	1.45 (1.44, 1.46)	1.24 (1.24, 1.25)	1.01 (1.00,1.02)	<0.001
Antidepressants (%)	13.17 (12.52, 13.84)	12.65 (11.66, 13.70)	13.76 (12.67, 14.93)	13.25 (12.25, 14.33)	13.01 (11.83, 14.28)	0.477
Anxiolytics, sedatives, and hypnotics (%)	6.78 (6.33, 7.27)	7.29 (6.54, 8.12)	6.78 (6.07, 7.56)	6.31 (5.56, 7.15)	6.75 (5.93, 7.68)	0.339

For continuous variables: survey-weighted mean (95% CI), the *p*-value was by survey-weighted linear regression (svyglm). For categorical variables: survey-weighted percentage (95% CI), the *p*-value was by survey-weighted Chi-square test (svytable).

Abbreviations: DM, diabetes mellitus; CVD: cardiovascular disease; BMI, body mass index; HbA1c, glycosylated hemoglobin; TC, total cholesterol; HDL, high-density lipoprotein; HTN, hypertension; PIR, poverty-income ratio (ratio of family income to poverty threshold); SD, standard deviation

Association between AC and depression

In the fully adjusted model, we observed a linear relationship between AC and depression (Figure 2). The results of the weighted multivariate logistic regression analysis are presented in Table 2. AC was positively correlated with depression in the crude model (odds ratio [OR]= 1.08, 95% confidence interval [CI]:1.06-1.11, *P* <0.001). A significant association between AC and depression was detected in

For peer review only

Models 1-3 after adjusting for confounders. In Model 3, all variables were adjusted; for every 1 unit increase in AC, the incidence of depression increased by 3% (OR = 1.03, 95% CI = 1.00–1.06, *P* = 0.039).

Table 2. Associations of the atherogenic coefficient with depression (n = 32,502), Weighted.

	**Crude Modela**		**Model 1b**		**Model 2c**		**Model 3d**	
	**OR (95% CI)**	***p*-value**	**OR (95% CI)**	***p*-value**	**OR (95% CI)**	***p*-value**	**OR (95% CI)**	***p*-value**
Per 1 increase	1.08 (1.06,1.11)	<0.001	1.13 (1.10,1.16)	<0.001	1.07 (1.04,1.10)	<0.001	1.03 (1.00,1.06)	0.039
Quartiles								
Q1 (AC: < 1.9310)	Reference [1]		Reference [1]		Reference [1]		Reference [1]	
Q2 (AC: 1.9310 to < 2.6695)	1.11 (0.96,1.30)	0.166	1.18 (1.01,1.38)	0.040	1.07 (0.91,1.25)	0.431	1.04 (0.89,1.22)	0.589
Q3 (AC: 2.6695 to < 3.6430)	1.30 (1.11,1.53)	0.001	1.48 (1.26,1.74)	<0.001	1.24 (1.07,1.45)	0.006	1.18 (1.02,1.38)	0.034
Q4 (AC: ≥ 3.6430)	1.41 (1.22,1.62)	<0.001	1.75 (1.50,2.03)	<0.001	1.32 (1.14,1.54)	<0.001	1.15 (0.99,1.33)	0.074
p for trend	<0.001		<0.001		<0.001		0.040	

Abbreviations: AC, atherogenic coefficient.

aModel 1: Adjusted for age, sex, and race/ethnicity.

bModel 2: Adjusted for the variables in Model 1 plus body mass index, poverty-income ratio, educational level, and marital status.

cModel 3: Adjusted for the variables in Model 2 plus hypertension, diabetes mellitus, alcohol intake, smoking status, physical activity, and glycosylated hemoglobin.

After adjusting for age, sex, race/ethnicity, BMI, PIR, educational level, marital status, HTN, DM, alcohol intake, smoking status, physical activity, and HbA1c compared with participants in the first quartile (AC< 1.9310), the second group (1.9310 to < 2.6695, OR = 1.04, 95% CI = 0.89– 1.22, *P* = 0.589), the third group (2.6695 to < 3.6430, OR = 1.18, 95% CI = 1.02– 1.38, *P* = 0.034), and the fourth group (≥ 3.6430, OR = 1.15, 95% CI = 0.99– 1.33, *P* = 0.074) had an increased prevalence of depression (*P* for trend was significant in all the models).

For peer review only

For peer review only - http://bmjopen.bmj.com/site/about/guidelines.xhtml

Furthermore, regarding the interaction between sex and the relationship between AC and depression, the relationship was more significant in females (OR = 1.07, 95% CI = 1.02–1.12, *P* for interaction = 0.027) (Table 3).

Table 3. Subgroup analysis of the effect of the atherogenic coefficient on depression (n = 32502), Weight.

Subgroup	Number of participants	OR (95% CI)	*P* for interaction
Sex, n (%)			0.027
Male	15954	1.05 (1.01, 1.10)	
Female	16548	1.07 (1.02, 1.12)	
Age, n (%)			0.375
20 – < 40	10857	1.07 (1.02, 1.12)	
40 – < 60	10632	1.04 (0.99, 1.09)	
≥ 60	11013	1.04 (0.97, 1.11)	
Race/ethnicity, n (%)			0.196
Non-Hispanic White	14112	1.03 (0.99, 1.08)	
Non-Hispanic Black	6713	1.06 (0.98, 1.15)	
Mexican American	5174	1.05 (0.97, 1.14)	
Other Hispanic	3109	1.12 (1.06, 1.19)	
Other race/multiple races	3394	1.10 (0.96, 1.26)	
BMI, kg/m2, mean (SD)			0.212
Low	10729	1.08 (1.00, 1.17)	
Middle	10733	1.06 (1.01, 1.12)	
High	10743	1.03 (0.99, 1.08)	
Hypertension, n (%)			0.949
Yes	13940	1.04 (1.00, 1.08)	
No	18562	1.06 (1.01, 1.12)	
DM, n (%)			0.670
Yes	6152	1.03 (0.98, 1.08)	
No	25761	1.07 (1.03, 1.11)	

Abbreviations: BMI, body mass index (calculated as weight, in kilograms, divided by the square of height, in meters); DM, diabetes mellitus; HTN, hypertension

DISCUSSION

This cross-sectional study showed an association between AC and depression in adults in the United States. After adjusting for covariates, a positive linear relationship was found between AC and depression.

For peer review only - http://bmjopen.bmj.com/site/about/guidelines.xhtml

The details of the mechanism explaining the relationship between AC and depression need to be further explored, and there may be several possible explanations. Lipids and the immune system interact with one another and have a regulatory effect on each other. Dysregulated inflammation promotes susceptibility to depression [23]. Studies have shown that inflammatory cytokines produced in the periphery enter the cells of the central nervous system and can affect neurotransmitters and neural circuits, producing behavioral symptoms of depression [24]. When T lymphocytes are activated, they not only participate in immune inflammation but also directly contribute to the development of depression when functionally impaired [25,26]. Lipid peroxidation and oxidation-specific epitopes are formed, and the levels of antioxidants such as glutathione, glutathione peroxidase, and coenzyme Q10 are reduced, resulting in or aggravating oxidative stress [27,28].

When lipids are abnormal, the inflammatory, oxidative, and nitrosative (IO&NS) pathway is further activated [29]. At this time, the increase in binding globin and high-sensitivity C-reactive protein (CRP) is accompanied by a significant increase in interleukin-6 (IL-6), tumor necrosis factor-α, interferon-γ, other pro-inflammatory cytokines, and immune inflammation [30]. These factors lead to defects in serotonin and melatonin through the kynurenine pathway, often considered one of the main causes of depression [31]. Activation of the IO&NS pathway leads to mitochondrial and subsequent cellular dysfunction [32]. Previous studies have linked mitochondrial dysfunction in various brain regions to depression [33].

Statins have also been shown to have antidepressant effects when co-prescribed with antidepressants [34]. Lowering the AC index of patients with mood disorders improves CVD outcomes [35]. CVD is a heart and blood vessel disease characterized by myocardial infarction, angina pectoris, heart failure, heart attack, and stroke [36]. Longer exposure to depression is associated with significantly increased CVD risk [37]. Factors contributing to the link between depression and cardiac outcome may include alterations in the autonomic nervous system, platelet receptors and function, coagulopathic factors, pro-inflammatory cytokines, endothelial function, neurohormonal factors, and genetic linkages [38]. At the same

For peer review only - http://bmjopen.bmj.com/site/about/guidelines.xhtml

time, patient compliance with antidepressant treatment is relatively poor [39]. Atherosclerosis is a chronic vascular inflammatory disease associated with oxidative stress and endothelial dysfunction [40]. Atherosclerosis is the underlying cause of CVD, and AC is a major indicator of atherosclerosis [41]. Our results suggest that AC may play a role in depression. AC may indicate the relationship between CVD and depression and a potential target and marker for treating depression or depression combined with CVD. The relevant mechanisms remain to be explored further.

Our study found increased odds of depression with increased AC in adults, demonstrating that controlling AC may be beneficial for preventing depression. Sex may affect this relationship. In the subgroup analysis (Table 3), we found a stronger relationship between AC and depression in females. The synergistic effect of estrogen on cognitive and emotional functions may underlie the association between ovarian hormone fluctuations and depression in females [42]. The induction of indoleamine 2, 3-dioxygenase, and deleterious effects of tryptophan catabolizing metabolites (TRYCATs) play a role in the pathophysiology of depression. Activation of IDO decreases plasma tryptophan levels and increases TRYCAT synthesis in depressed individuals. Females showed more IDO activation and TRYCAT production after immune challenge than males [43]. Therefore, this sex difference in immune dysregulation may contribute to higher levels of anxiety and depression experienced by females.

This study has some limitations. First, this was a cross-sectional study; therefore, we could not determine a causal relationship between AC and depression. Second, the PHQ-9 is a proven, simple, and effective tool for identifying the severity of depressive symptoms, but it is not a diagnostic tool for MDD. Third, the relationship we studied may have been influenced by other confounding factors, which we have not adjusted. Fourth, the differences in demographics and population characteristics in the United States may limit the generalizability of the findings to other countries or regions. Fifth, AC is associated with both depression and CVD, which may potentially affect the nervous system and mental health. At the same time, there are also uncontrollable variables such as lifestyle and regional culture. Finally,

For peer review only - http://bmjopen.bmj.com/site/about/guidelines.xhtml

although collinearity statistics did not find any significant collinearity, relationships that are beyond statistical *p*-values may exist.

CONCLUSIONS

Our research shows that higher AC levels in American adults are positively related to a higher prevalence of depression. Further studies are required to explore the underlying mechanisms and potential benefits of controlling AC levels in patients with depression.

For peer review only

Funding: This work was supported by the Shandong Province Traditional Chinese Medicine Science and Technology project key project Z-2023019.

Competing Interests: This research received no specific grant from any funding agency in the public, commercial, or not-for-profit sectors.

Author Contributions: Conceptualization, L Zhang, J Yin; methodology, L Zhang; software, J Yin; validation, H Sun, Y Liu and J Yang; formal analysis, L Zhang, J Yin, and H Sun; investigation, L Zhang; resources, L Zhang; data curation, H Sun; writing—original draft preparation, L Zhang; writing—review and editing, Y Liu and J Yang; visualization, J Yin; supervision, Y Liu and J Yang; project administration, L Zhang. All authors have read and agreed to the published version of the manuscript.

Acknowledgments: Thanks to the acupuncture-medicine team. Thanks to Prof. Liu and Prof. Yang for their careful teaching. Thanks to our colleagues for their help. Thanks to all who contributed to the manuscript.

Data Sharing Statement: The original contributions presented in the study are included in the article/supplementary material, further inquiries can be directed to the corresponding author.

Ethics Statement: The National Center for Health Statistics (NCHS) Research Ethics Review Board (ERB) authorized the NHANES study protocols. The data for this study were obtained from the NHANES database, no one was directly involved, and no additional ethical guidelines were required.

For peer review only - http://bmjopen.bmj.com/site/about/guidelines.xhtml

References

[1]McCarron RM, Shapiro B, Rawles J, et al. Depression. Ann Intern Med. 2021;174:ITC65–ITC80.

[2]Malhi GS, Mann JJ. Depression. Lancet. 2018;392:2299–2312.

[3]Stringaris A. Editorial: what is depression? J Child Psychol Psychiatry. 2017;58:1287–1289.

[4]Reeder N, Tolar-Peterson T, Bailey RH, et al. Food Insecurity and Depression among US Adults: NHANES 2005-2016. Nutrients. 2022;14:3081.

[5]Meng R, Yu C, Liu N, et al. Association of Depression With All-Cause and Cardiovascular Disease Mortality Among Adults in China. JAMA Netw Open. 2020;3:e1921043.

[6]Hao Y, Ge H, Sun M, et al. Selecting an appropriate animal model of depression. Int J Mol Sci. 2019;20:4827.

[7]Edinoff AN, Akuly HA, Hanna TA, st al. Selective Serotonin Reuptake Inhibitors and Adverse Effects: A Narrative Review. Neurol Int. 2021;13:387-401.

[8]Andrews B, Klein B, Van Nguyen H, et al. Efficacy of a Digital Mental Health Biopsychosocial Transdiagnostic Intervention With or Without Therapist Assistance for Adults With Anxiety and Depression: Adaptive Randomized Controlled Trial. J Med Internet Res. 2023;25:e45135.

[9]Milaneschi Y, Simmons WK, van Rossum EFC, et al. Depression and obesity: evidence of shared biological mechanisms. Mol Psychiatry. 2019;24:18–33.

[10]Able AA, Richard AJ, Stephens JM. TNFα Effects on Adipocytes Are Influenced by the Presence of Lysine Methyltransferases, G9a (EHMT2) and GLP (EHMT1). Biology (Basel). 2023;12:674.

[11]Otte C, Gold SM, Penninx BW, et al. Major depressive disorder. Nat Rev Dis Primers. 2016;2:16065.

[12]Beverly JK, Budoff MJ. Atherosclerosis: pathophysiology of insulin resistance, hyperglycemia, hyperlipidemia, and inflammation. J Diabetes. 2020;12:102–104.

[13]Mahdavi-Roshan M, Shoaibinobarian N, Noormohammadi M, et al. Inflammatory Markers and Atherogenic Coefficient: Early Markers of Metabolic Syndrome. Int J Endocrinol Metab. 2022;20:e127445.

For peer review only - http://bmjopen.bmj.com/site/about/guidelines.xhtml

[14]Nunes SO, Piccoli de Melo LG, Pizzo de Castro MR, et al. Atherogenic index of plasma and atherogenic coefficient are increased in major depression and bipolar disorder, especially when comorbid with tobacco use disorder. J Affect Disord. 2015;172:55–62.

[15]Tao SH, Ren XQ, Zhang LJ, et al. Association between common cardiovascular drugs and depression. Chin Med J (Engl). 2021;134:2656–2665.

[16]Walker AJ, Kim Y, Borissiouk I, et al. Statins: neurobiological underpinnings and mechanisms in mood disorders. Neurosci Biobehav Rev. 2021;128:693–708.

[17]Fain JA. NHANES. Diabetes Educ. 2017;43:151.

[18]Patel JS, Oh Y, Rand KL, et al. Measurement invariance of the patient health questionnaire-9 (PHQ-9) depression screener in U.S. adults across sex, race/ethnicity, and education level: NHANES 2005-2016. Depress Anxiety. 2019;36:813-823.

[19]Levis B, Benedetti A, Thombs BD, et al. Accuracy of Patient Health Questionnaire-9 (PHQ-9) for screening to detect major depression: individual participant data meta-analysis. BMJ. 2019;365:l1476.

[20]Brooks JM, Titus AJ, Bruce ML, et al. Depression and handgrip strength among U.S. adults aged 60 years and older from NHANES 2011-2014. J Nutr Health Aging. 2018;22:938–943.

[21]Levis B, Benedetti A, Thombs BD; DEPRESsion Screening Data (DEPRESSD) Collaboration. Accuracy of Patient Health Questionnaire-9 (PHQ-9) for screening to detect major depression: individual participant data meta-analysis. BMJ. 2019;365:l1476.

[22]Stekhoven DJ, Bühlmann P. MissForest—non-parametric missing value imputation for mixed-type data. Bioinformatics. 2012;28:112–118.

[23]Beurel E, Toups M, Nemeroff CB. The bidirectional relationship of depression and inflammation: double trouble. Neuron. 2020;107:234–256.

[24]Capuron L, Lasselin J, Castanon N. Role of adiposity-driven inflammation in depressive morbidity. Neuropsychopharmacology. 2017;42:115–128.

[25]Haapakoski R, Ebmeier KP, Alenius H, et al. Innate and adaptive immunity in the development of depression: an update on current knowledge and technological advances. Prog Neuropsychopharmacol Biol Psychiatry. 2016;66:63–72.

[26]Miller AH. Depression and immunity: a role for T cells? Brain Behav Immun. 2010;24:1–8.

For peer review only - http://bmjopen.bmj.com/site/about/guidelines.xhtml

[27]Bartolini Gritti B, Binder CJ. Oxidation-specific epitopes are major targets of innate immunity in atherothrombosis. Hamostaseologie. 2016;36:89–96.

[28]Maes M, Mihaylova I, Kubera M, et al. Increased plasma peroxides and serum oxidized low density lipoprotein antibodies in major depression: markers that further explain the higher incidence of neurodegeneration and coronary artery disease. J Affect Disord. 2010;125:287–94.

[29]Morris G, Puri BK, Walker AJ, et al. Shared pathways for neuroprogression and somatoprogression in neuropsychiatric disorders. Neurosci Biobehav Rev. 2019;107:862–882.

[30]Maes M, Ruckoanich P, Chang YS, et al. Multiple aberrations in shared inflammatory and oxidative & nitrosative stress (IO&NS) pathways explain the co-association of depression and cardiovascular disorder (CVD), and the increased risk for CVD and due mortality in depressed patients. Prog Neuropsychopharmacol Biol Psychiatry. 2011;35:769–783.

[31]Gałecki P, Talarowska M. Inflammatory theory of depression. Psychiatr Pol. 2018;52:437–447.

[32]Mezzaroba L, Alfieri DF, Colado Simão AN, et al. The role of zinc, copper, manganese and iron in neurodegenerative diseases. Neurotoxicology. 2019;74:230–241.

[33]Bansal Y, Kuhad A. Mitochondrial dysfunction in depression. Curr Neuropharmacol. 2016;4:610–618.

[34]Singh D, Lippmann S. Can statins diminish depression? Prim Care Companion CNS Disord. 2018;20:17br02169.

[35]Zhang Y, Chen Y, Ma L. Depression and cardiovascular disease in elderly: current understanding. J Clin Neurosci. 2018;47:1–5.

[36]Xu C, Liang J, Xu S, et al. Increased serum levels of aldehydes are associated with cardiovascular disease and cardiovascular risk factors in adults. J Hazard Mater. 2020;400:123134.

[37]Zhang Y, Li X, Chan VKY, et al. Depression duration and risk of incident cardiovascular disease: A population-based six-year cohort study. J Affect Disord. 2022;305:188-195.

[38]de Jonge P, Rosmalen JG, Kema IP, et al. Psychophysiological biomarkers explaining the association between depression and prognosis in coronary artery

For peer review only - http://bmjopen.bmj.com/site/about/guidelines.xhtml

patients: a critical review of the literature. Neurosci Biobehav Rev. 2010;35:84–90.

[39]Shrestha Manandhar J , Shrestha R , Basnet N , et al. Study of Adherence Pattern of Antidepressants in Patients with Depression. Kathmandu Univ Med J (KUMJ). 2017;15:3-9.

[40]Marchio P, Guerra-Ojeda S, Vila JM, et al. Targeting early atherosclerosis: a focus on oxidative stress and inflammation. Oxid Med Cell Longev. 2019;2019:8563845.

[41]Nitsa A, Toutouza M, Machairas N, et al. Vitamin D in cardiovascular disease. In Vivo. 2018;32:977–981.

[42]Albert KM, Newhouse PA. Estrogen, stress, and depression: cognitive and biological interactions. Annu Rev Clin Psychol. 2019;15:399–423.

[43]Maes M, Leonard BE, Myint AM, et al. The new '5-HT' hypothesis of depression: cell-mediated immune activation induces indoleamine 2,3-dioxygenase, which leads to lower plasma tryptophan and an increased synthesis of detrimental tryptophan catabolites (TRYCATs), both of which contribute to the onset of depression. Prog Neuropsychopharmacol Biol Psychiatry. 2011;35:702–721.

FIGURE LEGENDS

Figure 1. Flowchart for inclusion of study participants.

Figure 2. Association between AC and depression in US adults (n=4759). The black vertical line on the horizontal axis represents the AC distribution, the red line represents the best fit, and the difference between the dashed lines represents the 95% confidence interval. The data was adjusted for age, sex, race/ethnicity, body mass index, poverty-income ratio, educational level, marital status, hypertension, diabetes mellitus, alcohol intake, smoking status, physical activity, and glycosylated hemoglobin.

For peer review only - http://bmjopen.bmj.com/site/about/guidelines.xhtml

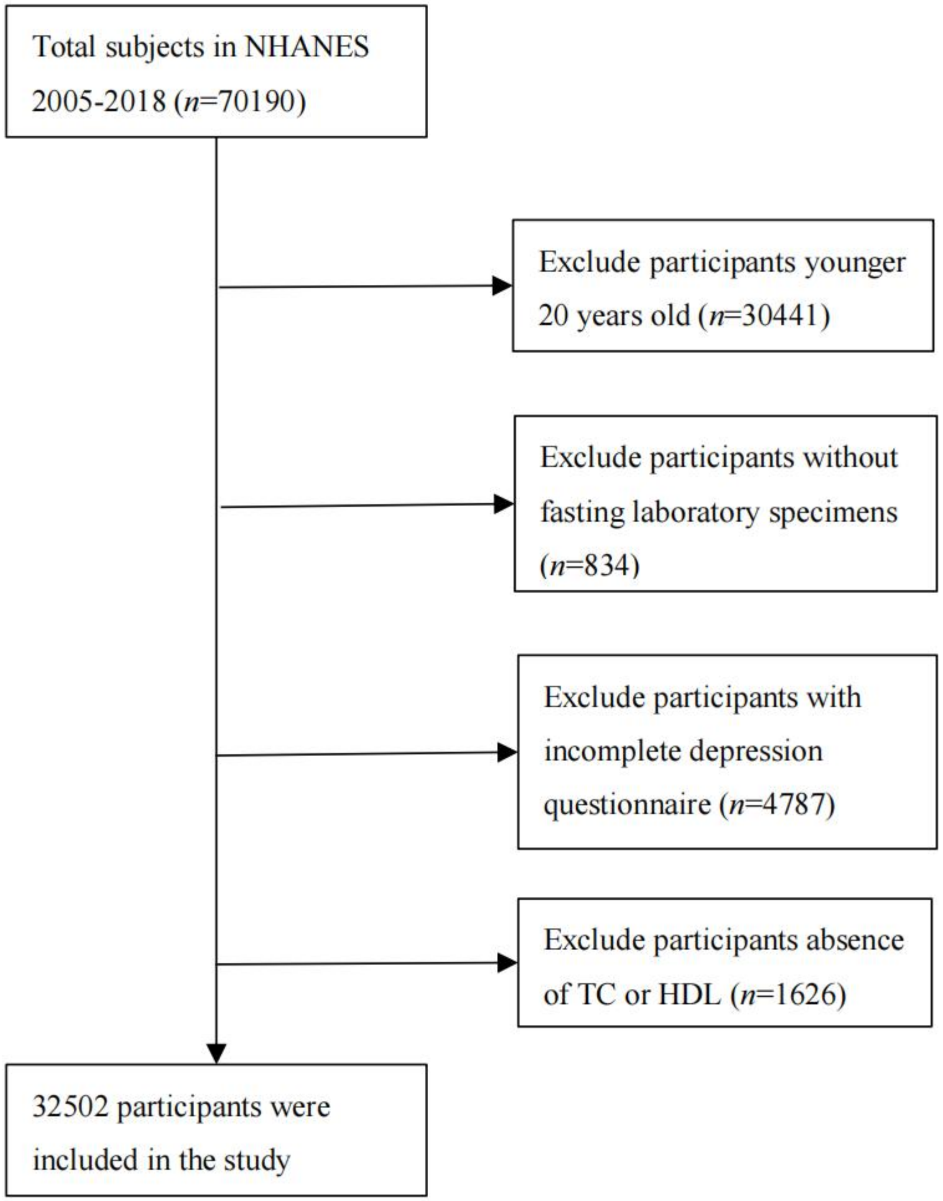

Figure 1. Flowchart for inclusion of study participants.

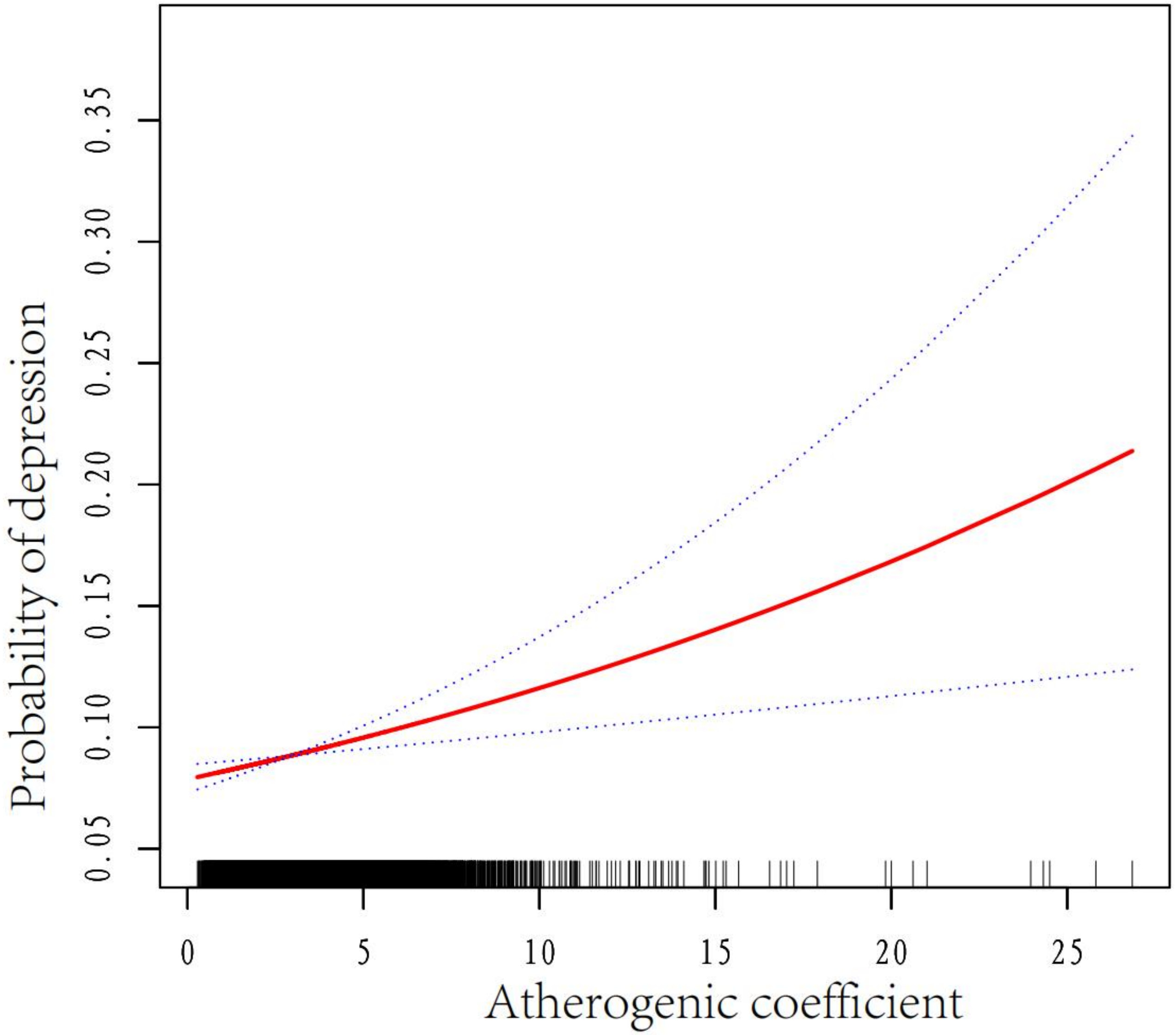
Probability of depression
0.05
0.10
0.15
0.20
0.25
0.30
0.35
0
5
10
15
20
25
Atherogenic coefficient

Table S1. Missing covariates of study participants (n = 32502)

Variable	Number of patients (% missing)
Age	0 (0%)
Sex	0 (0%)
Race/ethnicity	0 (0%)
Educational level	23 (0.07%)
Marital status	16 (0.04%)
Poverty-income ratio	2767 (8.51%)
Body mass index	297 (0.91%)
Alcohol intake	4661 (14.34%)
Smoking status	17 (0.05%)
Physical activity	4102 (12.62%)
Hypertension	0 (0%)
Diabetes mellitus	589 (1.81%)
Glycosylated hemoglobin	58 (0.18%)

For peer review only

Table S2. Absence of atherogenic coefficient in adults

**Age (y)**	**Missing number (n)**	**Total number (n)**	**Proportion (%)**
20-29	729	6029	12.09
30-39	665	6044	11.00
40-49	527	6060	8.70
50-59	524	5691	9.21
60-69	575	5974	9.63
70-79	425	3730	11.39
≥80	446	2330	19.14

Table S3. Threshold effect analysis for association of atherogenic coefficient with depression

**Outcomes**	**Depression**	***P*-value**
Model 1, β(95%)		
Linear effort model	1.04(1.02,1.07)	0.002
Model 2, β(95%)		
Infection point (K)	1.2	
K ＜1.2	0.54 (0.29,1.03)	0.059
1.2 ＞K	1.05 (1.02,1.08)	＜0.001
LLR	0.051	

Table S4. Results of collinearity detection

Model		Unstandardized Coefficients		Standardized Coefficients	t	Significance	Collinearity Statistics	
		B	Standard Error				Tolerance	VIF
1	Constant	-0.067	0.01		-6.668	0		
	AC	0.002	0.002	0.01	1.089	0.276	0.153	6.524
	HDL cholesterol	-0.002	0.006	-0.003	-0.349	0.727	0.193	5.177
	TC	-0.001	0.002	-0.002	-0.312	0.755	0.269	3.723
	HbA1c	0.001	0.001	0.004	0.973	0.33	0.936	1.068

Dependent Variable: Depression.

Abbreviations: AC, atherogenic coefficient; HDL, high-density lipoprotein; TC, total cholesterol; HbA1c, glycosylated hemoglobin.

For peer review only

STROBE 2007 (v4) Statement—Checklist of items that should be included in reports of *cross-sectional studies*

**Section/Topic**	**Item #**	**Recommendation**	**Reported on page #**
**Title and abstract**	1	(*a*) Indicate the study's design with a commonly used term in the title or the abstract	2
		(*b*) Provide in the abstract an informative and balanced summary of what was done and what was found	2
**Introduction**			
Background/rationale	2	Explain the scientific background and rationale for the investigation being reported	3
Objectives	3	State specific objectives, including any prespecified hypotheses	4
**Methods**			
Study design	4	Present key elements of study design early in the paper	
Setting	5	Describe the setting, locations, and relevant dates, including periods of recruitment, exposure, follow-up, and data collection	
Participants	6	(*a*) Give the eligibility criteria, and the sources and methods of selection of participants	4
Variables	7	Clearly define all outcomes, exposures, predictors, potential confounders, and effect modifiers. Give diagnostic criteria, if applicable	5-6
Data sources/ measurement	8*	For each variable of interest, give sources of data and details of methods of assessment (measurement). Describe comparability of assessment methods if there is more than one group	5-6
Bias	9	Describe any efforts to address potential sources of bias	*6-7*
Study size	10	Explain how the study size was arrived at	4
Quantitative variables	11	Explain how quantitative variables were handled in the analyses. If applicable, describe which groupings were chosen and why	5-6
Statistical methods	12	(*a*) Describe all statistical methods, including those used to control for confounding	6-7
		(*b*) Describe any methods used to examine subgroups and interactions	5
		(*c*) Explain how missing data were addressed	7
		(*d*) If applicable, describe analytical methods taking account of sampling strategy	
		(*e*) Describe any sensitivity analyses	
**Results**			

For peer review only

Participants	13*	(a) Report numbers of individuals at each stage of study—eg numbers potentially eligible, examined for eligibility, confirmed eligible, included in the study, completing follow-up, and analysed	7
		(b) Give reasons for non-participation at each stage	
		(c) Consider use of a flow diagram	7
Descriptive data	14*	(a) Give characteristics of study participants (eg demographic, clinical, social) and information on exposures and potential confounders	4
		(b) Indicate number of participants with missing data for each variable of interest	
Outcome data	15*	Report numbers of outcome events or summary measures	7
Main results	16	(*a*) Give unadjusted estimates and, if applicable, confounder-adjusted estimates and their precision (eg, 95% confidence interval). Make clear which confounders were adjusted for and why they were included	5-6
		(*b*) Report category boundaries when continuous variables were categorized	
		(*c*) If relevant, consider translating estimates of relative risk into absolute risk for a meaningful time period	
Other analyses	17	Report other analyses done—eg analyses of subgroups and interactions, and sensitivity analyses	12
**Discussion**			
Key results	18	Summarise key results with reference to study objectives	13-14
Limitations	19	Discuss limitations of the study, taking into account sources of potential bias or imprecision. Discuss both direction and magnitude of any potential bias	15
Interpretation	20	Give a cautious overall interpretation of results considering objectives, limitations, multiplicity of analyses, results from similar studies, and other relevant evidence	
Generalisability	21	Discuss the generalisability (external validity) of the study results	15
**Other information**			
Funding	22	Give the source of funding and the role of the funders for the present study and, if applicable, for the original study on which the present article is based	15

*Give information separately for cases and controls in case-control studies and, if applicable, for exposed and unexposed groups in cohort and cross-sectional studies.

Note: An Explanation and Elaboration article discusses each checklist item and gives methodological background and published examples of transparent reporting. The STROBE checklist is best used in conjunction with this article (freely available on the Web sites of PLoS Medicine at http://www.plosmedicine.org/, Annals of Internal Medicine at http://www.annals.org/, and Epidemiology at http://www.epidem.com/). Information on the STROBE Initiative is available at www.strobe-statement.org.

For peer review only
